# Collective privacy recovery: Data-sharing coordination via decentralized artificial intelligence

**DOI:** 10.1093/pnasnexus/pgae029

**Published:** 2024-01-22

**Authors:** Evangelos Pournaras, Mark Christopher Ballandies, Stefano Bennati, Chien-fei Chen

**Affiliations:** School of Computing, University of Leeds, Leeds LS2 3JT, UK; Computational Social Science, ETH Zurich, Zurich 8092, Switzerland; Computational Social Science, ETH Zurich, Zurich 8092, Switzerland; Institute for a Secure and Sustainable Environment, University of Tennessee, Knoxville, TN 37996, USA

## Abstract

Collective privacy loss becomes a colossal problem, an emergency for personal freedoms and democracy. But, are we prepared to handle personal data as scarce resource and collectively share data under the doctrine: as little as possible, as much as necessary? We hypothesize a significant privacy recovery if a population of individuals, the data collective, coordinates to share minimum data for running online services with the required quality. Here, we show how to automate and scale-up complex collective arrangements for privacy recovery using decentralized artificial intelligence. For this, we compare for the first time attitudinal, intrinsic, rewarded, and coordinated data sharing in a rigorous living-lab experiment of high realism involving >27,000 real data disclosures. Using causal inference and cluster analysis, we differentiate criteria predicting privacy and five key data-sharing behaviors. Strikingly, data-sharing coordination proves to be a win–win for all: remarkable privacy recovery for people with evident costs reduction for service providers.

**Significance Statement**

Privacy loss remains a long-standing problem, undermining personal freedoms and democracy. So far, data-sharing choices fail to balance privacy preservation and quality of online services based on shared data. We show that without a collective arrangement of what data to share, to whom and for what purpose, significant privacy is compromised, while business costs and risks increase. For the first time, we bridge this coordination gap via a novel and scalable decision support using decentralized trustworthy artificial intelligence. Coordination empowers communities to share data under the doctrine “as little as possible, as much as necessary.” With a comprehensive understanding of criteria that influence data-sharing decisions, we set foundations for a long-awaited renaissance of privacy in the digital era.

## Introduction

**Fig. 1.**
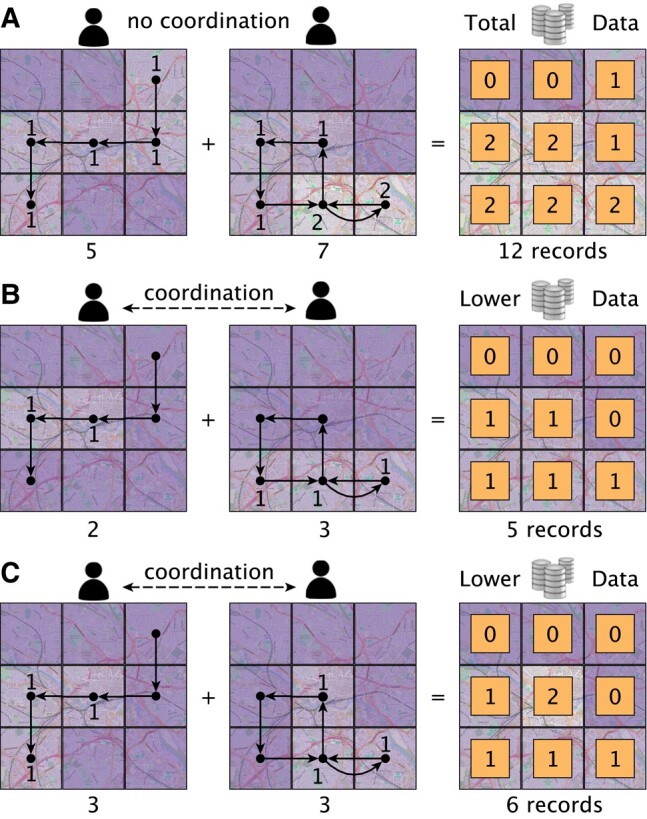
A motivational example on how coordinated data sharing recovers significant privacy. Individuals coordinate to collectively choose where to share and not share their location. A) Existing data-sharing status quo. Two individuals (e.g. drivers) move within an area of 3×3=9 possible locations (e.g. points of interest). By continuously sharing their Global Positioning System (GPS) location (default), they reveal sensitive information that can even disclose their identity (≥4 location records (2)). But, here we also show that these shared data are in practice redundant in several practical scenarios. For instance, B) determining the highest traffic density areas or C) prioritizing accurate traffic density estimation in the city center over the periphery can be both achieved with half (or even lower) the original data, while reducing privacy risks (<4 location records (2)) with fairer data sharing contributions among the individuals.

Control over sharing or giving access to personal data from pervasive devices, such as smartphones, turns out to be complex, involving critical decisions for privacy with impact on society. How to run data-intensive online services to improve everyday life without compromising personal values and freedoms? For instance, four apps (1) or spatiotemporal points (2) are enough to identify 91.2 and 95% of individuals. In practice, the data-sharing doctrine “as little as possible, as much as necessary” has not yet found a systematic and scalable applicability. The quality of online services is often a result of collective data-sharing decisions made by individuals consuming these services, for instance, traffic predictions using mobility data (2, 3). To achieve a minimum quality of service for a population of individuals while maximizing their privacy, a collective arrangement (i.e. coordination) of their data-sharing decisions is required to minimize both excessive and insufficient levels of data sharing (4–6). Figure 1 provides an illustrative example of the huge under-explored potential of coordinated data sharing for privacy.

### Privacy loss is a coordination deficit with large impact

Although a recent survey finds a 58% of individuals willing to balance data sharing case-by-case (7), it proves cognitively and computationally hard to achieve (8) even when using state-of-the-art privacy preservation techniques such as differential privacy (9, 10), secure multiparty computation (11), and k-anonymization (6, 12). The absence, failure, or inefficiency of this coordination exhibit a tragedy of the (data sharing) commons, making privacy easier to compromise than quality of service. As a result, studies show that 90% of individuals tend to give up privacy of their data, often without any added value (8), although 76% intend to protect it (13, 14). This insight is fundamental to several studies on the willingness to accept rewards for giving up privacy or willingness to pay a cost for preserving privacy (8, 15–17). Implications of giving up excessive personal data include energy-intensive and expensive data centers with unprocessed data growing faster than Moore’s law predictions, stress and anxiety, algorithmic biases, discrimination, censorship, and influence of election results (8, 18–22). Therefore, establishing a coordinated data sharing is a collective action for the recovery of privacy with an immense impact on the environment, health, society, and democracy.

### How to make coordinated data sharing feasible

**Fig. 2.**
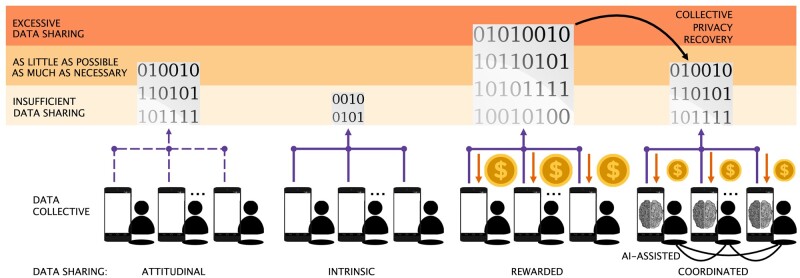
Tragedies of data-sharing commons showing a coordination deficiency. We hypothesize that while individuals may rationally intend to share a sufficient level of data, they end sharing intrinsically an insufficient level. If rewarded, data sharing is excessive with significant privacy loss. When coordination is introduced via a trustworthy AI-based decision-support system, significant privacy is recovered while achieving the desired quality of service. These studied hypotheses are formalized into four data-sharing conditions: (i) attitudinal, (ii) intrinsic, (iii) rewarded, and (iv) coordinated.

While privacy control is found essential for 82% of individuals in an earlier study (7, 23), so is convenience for 63%. The computational and communication load to coordinate data-sharing decisions at scale is overwhelming for humans alone. Instead, a scalable decision support can be provided by interactive personal assistants using cooperative artificial intelligence (AI) to cope with such complexity (24). These assistants can run on (mobile) devices of individuals who form a community (i.e. data collective) to consume an online service that relies on data they share as a result of a collective arrangement. In practice, the remote personal assistants interact in the background to coordinate *how much and what data to share, to which data collector, and for what purpose* (see Figs. 2 and 9a). These multiagent interactions and calculations self-organize into fully decentralized unsupervised learning process (25) that optimizes data-sharing efficiency: maximizing quality of service and minimizing privacy cost. Compared to other AI approaches for personalized privacy assistants (26) applied to legal document analytics (27) and pervasive devices (28), this decision-support system is itself privacy-preserving and does not rely on any centralized third party (selected “outstanding” by UNESCO IRCAI (29)). Therefore, the interactive personal assistants are trustworthy by design to serve as the privacy enabler of the data collective. This comes in stark contrast to the mainstream use of supervised AI algorithms that often require large concentrations of sensitive personal data for training (18, 22, 30). The proposed decision-support system can also operate as a trustworthy collective access control to local data by federated learning algorithms to train models in a privacy-preserving way (30, 31).

### Hypotheses for understanding data-sharing conditions

The overarching aim of this study is to assess the capacity of this novel AI-based system to steer the data collective into more efficient and privacy-preserving trajectories for data sharing. Figure 2 illustrates the main studied hypotheses. These hypotheses are formalized into four experimental conditions for data sharing under repeated measures (within-subjects design). They are rigorously compared with each other under high realism in a novel living-lab experiment, see Living-lab experimental design section, Figs. 8 and 9. Over 27,403 high-quality records of real data-disclosure decisions are collected by a novel platform developed for this purpose (see the Technical infrastructure section). It encompasses a smartphone app, a server to collect experimental data as well as a web portal with which the involved data collectors can access the shared data according to the privileges that participants give. The four studied experimental conditions shown in Fig. 2 are the following:

1. **Attitudinal data sharing** assesses how privacy-sensitive individuals perceive each of the 3 criteria×4 elements/  criterion=12 data-sharing elements, see Table S4, Questions B.9 to B.12 in supplementary material.
2. **Intrinsic data sharing** assesses actual decisions made for voluntarily data sharing (without rewards) in a complete factorial design of 4 sensors×4 collectors×4 contexts=64 data-sharing scenarios (see Fig. S3b).
3. **Rewarded data sharing** introduces an accumulated privacy-reward balance that individuals initially influence with their choices over the 64 data-sharing scenarios (see Fig. 9b). The built up balance can be further calibrated by making on-demand and repeated (unlimited within 24 h) choices among the 64 data-sharing scenarios retrieved automatically. Each retrieved scenario is calculated to improve the individual’s choice: privacy or rewards, see Fig. 9c. To account for threats to validity and trace any order effects, this experimental condition is repeated twice (2×24 h) by clearing the privacy-reward balance and collecting new data from sensors to share (Fig. 8b). To challenge privacy preservation, the rewards are personalized by inflating and deflating the amounts based on each individual’s privacy perception derived from attitudinal data sharing, see Section S1. This design choice is also expected to engage participants more effectively by rewarding the data-sharing scenarios fairly, according to participants’ personal values (8), while discouraging dropouts.
4. **Coordinated data sharing** relies on the AI-based personal assistants. They use the intrinsic and rewarded data-sharing levels as discrete options to choose from (ex post condition). Each assistant makes an optimized choice among these so that the combination of all collective choices recovers the privacy loss of the rewarded data sharing, while reducing the *mismatch* (discrepancy/fitness measure) between the shared and the required data by a service provider. This is a quality-of-service indicator that finds general applicability in adaptive sensor selection and flexible data fusion for several smart city and industrial applications (32–34). Matching can also be applied by a coordinated data collective to preserve *k*-anonymity in a bottom-up way, i.e. no more than *k* individuals share any combination of personal data (6, 12, 35).

### Smartphone sensor data play a pivotal role on privacy

This article studies sharing of smartphone sensor data with five discrete choices to choose from (uniform sampling of 100 to 0% of sensor data with a step of 25%), see Fig. 9c. These choices are applied to the total sensor data collected with a fixed frequency of 30 s (100% of data). This is a simple and general discrete-choice model that serves the complexity of the experiment. It can be extended to more complex spatiotemporal models as outlined in the Discussion section. The study of smartphone sensor data is particularly impactful for both privacy and quality of online services. Sensor fusion has a paramount role in applications of smart homes, grids, and transportation (32). There is evidence that smartphone app developers delegate privacy to end-users as the former face challenges in providing privacy solutions at the design and implementation phase (36). In practice though, it is the powerful data intermediaries that leverage the terms of data-sharing agreements (1, 7). Sharing smartphone sensor data can be regulated via privacy-protection mechanisms with a natural utility-driven interpretation (buy–sell) such as differential privacy (5). Given the symbiotic relationship of individuals with their smartphones, capturing high-dimensional and diverse sensor data for different application scenarios, the study comes with a universal scope on privacy.

### A novel approach to understanding data-sharing decisions

The performed living-lab experiment is the first of its kind: (i) It brings together all four data-sharing conditions for comparison, including the novel one of coordinated data sharing. This is distinguished from earlier survey studies and empirical observations focusing on the two dimensions of intentions vs. behavior that comprise the privacy paradox (37, 38). (ii) The experimental design uses mixed modalities to achieve rigor within a controlled lab environment as well as realism, scale and external validity by tracing behavior out of the lab using a smartphone platform developed for this purpose (see the Technical infrastructure section). (iii) The 4×4×4 factorial design results in 64 data-sharing scenarios (see Fig. 9a). They involve the three data-sharing criteria that model the involved trust (data collectors) and risks (data type and context), and they are the ones that explain malleable data-sharing behaviors (8, 15, 39). This large spectrum comes in contrast to earlier experiments and field tests made within a context and involving a specific data-sharing scenario such as online social lending (40), crowdfunding (41), and commerce (15, 17, 42, 43).

## Results

Three key results are illustrated in this article: (i) Coordinated data sharing is efficient—it recovers privacy for people and reduces costs for service providers. This is by accessing less but better quality of data compared to rewarded data sharing in which individuals tend to share excessive/unnecessary data. (ii) Data collector and context are the most important criteria with which individuals makes data-sharing choices. For rewarded choices with privacy loss though, the type of shared data becomes the most important criterion. (iii) Individuals exhibit five key group-behavior changes from intrinsic to rewarded data sharing. They are stable, yet reinforcing.

### Coordinated data sharing recovers privacy and lowers costs

**Fig. 3.**
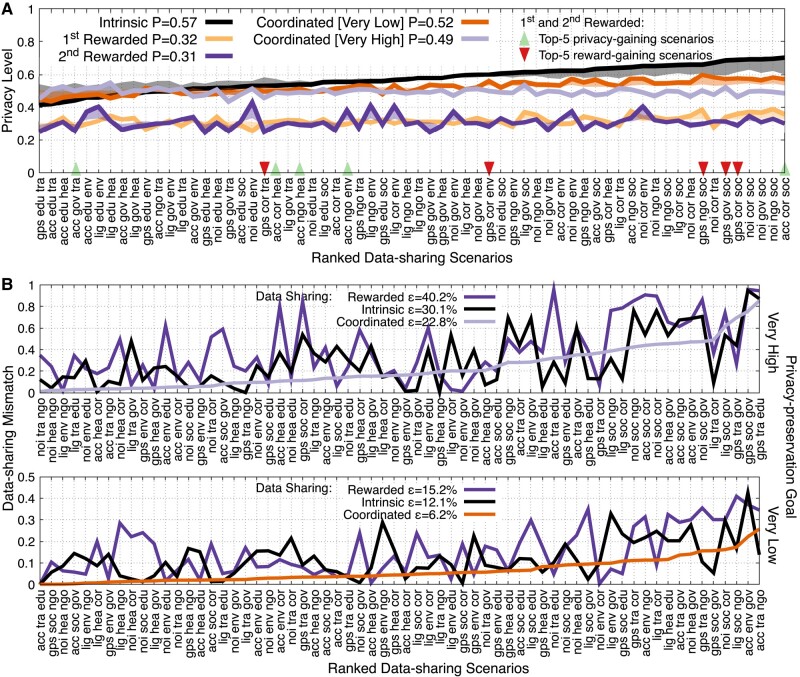
Coordinated data sharing over the 64 data-sharing scenarios shows higher efficiency than intrinsic and rewarded data sharing. A) Privacy (*P*, mean normalized data-sharing level) sorted from lowest to highest according to intrinsic data sharing. B) Data-sharing mismatch (ε, absolute error of standardized signals) between three data-sharing conditions and the privacy-preservation goal signals of very high and very low. Values are sorted from lowest to highest mismatch according to coordinated data sharing.

**Fig. 4.**
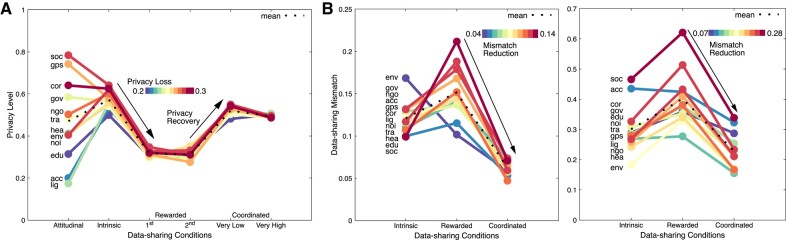
Coordination recovers significant privacy, while improving quality of data by lowering data-sharing mismatch. Privacy and data-sharing mismatch levels shown for different sensors, collectors and contexts under intrinsic, rewarded, and coordinated data sharing. The 12 lines are ranked according to the privacy loss (intrinsic−first rewarded data sharing) and mismatch reduction (first rewarded data sharing−coordinated). A) Privacy level, including the attitudinal data sharing. B) Data-sharing mismatch for the privacy-preservation goal signals of very low (left) and very high (right).

The privacy level and data-sharing quality (mismatch) are shown in Fig. 3 for the 64 data-sharing scenarios and the different experimental conditions. Figure 4 aggregates these measurements for each of the four sensors, data collectors, and contexts. The shaded areas in Fig. 3a illustrate the expected privacy level. It is derived by the mean privacy level of the sensor, collector, and context that comprise each data-sharing scenario (see the Privacy calculations for sensors, collectors, and contexts section for exact calculations).

**Fig. 5.**
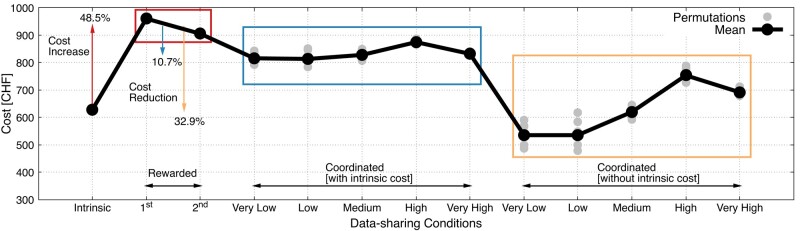
Coordinated data sharing reduces data-collection cost 10.7–32.9% compared to rewarded data sharing. This cost is comparable to intrinsic data sharing. Rewarded data sharing results in excessive data with 48.5% higher cost than intrinsic data sharing. Coordinated data sharing is calculated with and without the intrinsic cost. The light (gray) points represent the random permutations of the initial conditions in the optimization process.

The key observations are summarized as follows: (i) Coordinated data sharing results in significant privacy recovery (Figs. 3a and 4a) as well as more efficient data sharing (Fig. 3b and 4b) at a lower cost for service providers (Fig. 5). (ii) Intrinsic data sharing positively correlates to attitudinal data sharing but has a narrower range (Fig. 4a). (iii) Consecutive rewarded data sharing results in significant (and similar) privacy loss via, though, different data-sharing choices (Figs. 3a and 4a). (iv) The privacy loss, rather than the privacy level, under rewarded data sharing is correlated to the perceived privacy sensitivity (Fig. 4a). (v) Individuals improve their privacy by sharing data with lower privacy sensitivity than when improving rewards, while they keep sharing data to privacy-intrusive collectors under privacy-intrusive contexts (Fig. 3a).

#### Coordinated data sharing for efficiency and privacy recovery

Figure 3b illustrates the mismatch (absolute error) between a privacy-goal signal (very low and very high privacy preservation) and the aggregated data-sharing choices made via the AI approach (both standardized). Coordinated data sharing has a lower average mismatch than intrinsic and rewarded data sharing for both goal signals: 22.8<30.1<40.2% for very high and 6.2<12.1<15.2% for very low privacy preservation, respectively. With the very high privacy-preservation goal, matching is harder as out of three data-sharing plans to choose from, there is mainly one (intrinsic) containing data-sharing choices with high privacy preservation. On the contrary, with the very low privacy-preservation goal, mismatch is minimal by combining data-sharing plans from both the first and second rewarded data-sharing conditions. This trend is also confirmed in the other three privacy-goal signals (see Fig. S11, Section S8). For the very low and very high privacy-preservation goal, health (4.7, 16.5%) and noise (5.7, 16.6%) show a low mismatch on average, while government (7.3, 32.3%) and social networking (7.1, 33.8%) show a high one, see Fig. 4b. Via coordinated data sharing, social networking shows the highest mismatch reduction of 66.6 and 45.5% under the very low and very high privacy privacy-preservation goals. The overall average privacy recovery from rewarded to coordinated data sharing is 77%. These results demonstrate the unprecedented potential of coordinated data sharing to protect privacy, while retaining a data-sharing efficiency (see also Fig. S12, Section S9 illustrating different privacy-recovery valuations). Coordinated data sharing operates close to intrinsic data sharing with a minor (but significant: $t(63)=9.64,p=1.00\times 10^{-5}$ for the very low and $t(63)=7.81,p=1.00\times 10^{5}$ for the very high privacy-preservation goal) additional privacy sacrifice that is a benefit for data-sharing efficiency and as a result, the data collective as a whole.

#### Coordinated data sharing reduces data-collection costs

Figure 5 shows the incurred data-collection costs. The monetary cost of the first and second rewarded data sharing for data collectors is 960.18 CHF and 905.14 CHF, respectively. This cost is higher than the monetary value of the data shared intrinsically, which is 628.22 CHF. Strikingly, the cost of coordinated data sharing is on average 832.56 CHF (σ=15.93), which is on average 10.7% lower than the rewarded data sharing. These costs include the monetary value of intrinsic data sharing. If this value is excluded assuming that these data are shared for free (as happened in the experiment), the cost drops further down to 626.77 CHF, which is on average 32.9% lower than rewarded data sharing. It is remarkable that the monetary value of coordinated data sharing is similar to the one of intrinsic; however, it yields data of higher utility for service providers. As a result, coordinated data sharing is a win–win for all: lower data-collection costs for service providers, higher quality of service via improved data-sharing efficiency and significant privacy recovery for the participants of the data collective.

#### Attitudinal-intrinsic data sharing

Privacy preservation under intrinsic data sharing is 21.7% higher than the perceived privacy (Fig. 4a). While this difference is not significant (t(11)=−2.07,p=0.06), the privacy levels between the 12 elements of attitudinal and intrinsic data sharing are positively correlated (R=0.63,t(10)=2.54,p=0.029), despite the significant drop of 95.3% in the dispersion (variance). This result shows that data sharing operates in a narrower decision space than the perceived privacy. Social networking (0.78, 0.64) and corporation (0.64, 0.62) come with both high privacy sensitivity and preservation, while education (0.31, 0.5) and accelerometer (0.2, 0.53) show low privacy sensitivity and preservation.

#### Intrinsic-rewarded data sharing

Under the two rewarded data-sharing conditions, participants clearly give up privacy by 44% ($t(63)=-31.35,p=1.00\times 10^{-5}$) and 45.9% ($t(63)=-25.49,p=1.00\times 10^{-5}$), respectively (Fig. 3a, see also Fig. S9a and b in Section S6). The privacy level of intrinsic data sharing for the different sensors, collectors and contexts is correlated to the one of the first rewarded data sharing ($R=0.53,t(62)=4.99,p=5.00\times 10^{-6}$) but not to the one of the second rewarded data sharing (R=0.12,t(62)=0.94,p=0.79). Consecutive rewarded data sharing results in equivalent privacy preservation (t(63)=−1.22,p=0.23); nevertheless, this effect appears via different choices made within the data-sharing scenarios (R=0.033,t(62)=0.26,p=0.79).

#### Attitudinal-rewarded data sharing

Rewarded participants sacrifice privacy by 32.4% (t(11)=2.72,  p=0.013) and 34% (t(11)=2.85,p=0.009) compared to attitudinal data sharing (Fig. 3a). The privacy level under the two rewarded data-sharing conditions is not correlated to the perceived privacy sensitivity (attitudinal) of the different sensors, collectors and contexts (R=0.36,t(10)=1.22,p=0.24 and R=−0.39,t(10)=1.53,p=0.15 in Fig. 4a). Striking, though, it is the privacy loss (intrinsic-rewarded data sharing) that correlates to attitudinal data sharing (R=0.64,t(10)=2.64,p=0.025, R=0.77,t(10)=3.82,  p=0.0033).

#### Which data-sharing scenarios improve privacy and rewards?

Under rewards, data-sharing scenarios are automatically retrieved to fulfill participants’ goal, i.e. data-sharing options with the highest improvement of privacy or rewards, see Fig. 9. Figure 3a marks the top-5 scenarios that result in the highest mean privacy and reward gain (all ranked scenarios are presented in Fig. S8 and Table S10). The most highly privacy-gaining scenarios involve nonprivacy-sensitive sensor data such as accelerometer, which are shared though with privacy-intrusive data collectors and contexts such as social networking and corporation. In contrast, the most highly reward-gaining scenarios involve privacy-sensitive sensor data such as GPS, which are also shared with the privacy-intrusive data collectors and context of social networking and corporations. These observations reveal the following: Individuals improve privacy or rewards by sharing data under privacy-sensitive contexts to privacy-intrusive collectors. Nonetheless, compared to improving rewards, individuals change to sharing data with lower privacy sensitivity when improving their privacy.

### Rewarded individuals better distinguish data than collectors/contexts

**Fig. 6.**
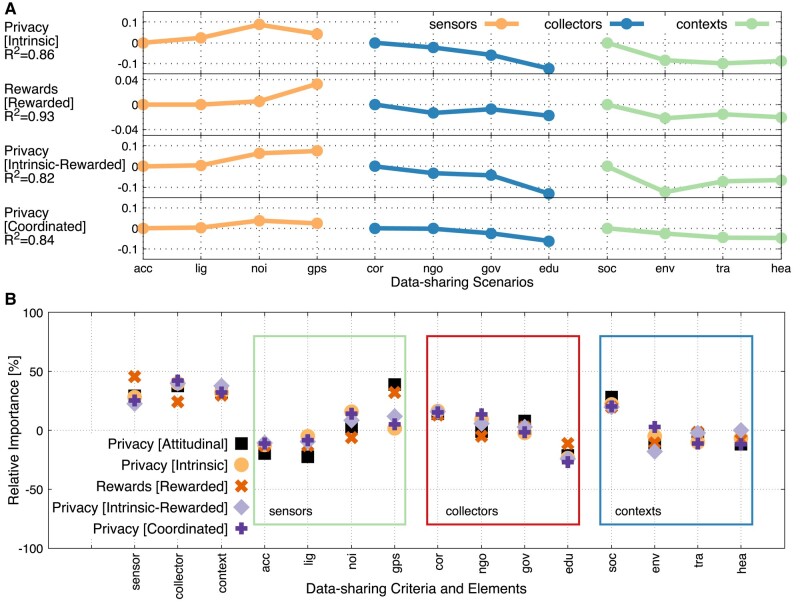
Rewarded individuals, who share data shift the importance from collectors and contexts to data. Via a conjoint analysis, four multiple linear regression models are compared. They explain how the different data-sharing criteria and elements influence different key data-sharing behaviors. A) Coefficients of the different regression models. The type of sensor data contributes positively to privacy preservation and rewards gain. Data collectors and context contribute negatively to privacy preservation and rewards gain. B) The relative importance (partworth utilities) of the data-sharing criteria and elements (relative within each criterion) derived from the different regression models of conjoint analysis and the perceived privacy sensitivity. The data collector is the most important criterion for the models based on privacy. In contrast, the sensor type is the most important criterion for the model based on rewards gain.

Here, we study the causal link between the data-sharing criteria/elements (independent variables) and the privacy/reward gains (dependent variables) in different experimental conditions. Four explanatory models based on a conjoint analysis are outlined in Causal inference with conjoint analysis section. Figure 6a illustrates the regression coefficients of the models, while Fig. 6b shows the relative importance of the data-sharing criteria and their elements calculated from these coefficients. All models come with $R^{2}>0.8$ and with statistically significant values of relative importance (p<0.05) for the vast majority of data-sharing elements as shown in Table S13, Section S11. Figure 6b also shows the perceived relative importance derived from the self-reported entry survey questions.

The data collector is the most important criterion (40.73% on average, Fig. 6b) for all models that predict privacy, and this criterion explains privacy loss (Fig. 6a). Context follows with a 33.91% of importance explaining privacy loss, while sensor type shows the lowest importance of 25.36%, explaining the privacy gains. The consistency of these three privacy models reveals the following: the data collectors to whom individuals share data determine to a high extent (i) the privacy level under intrinsic or coordinated data sharing and (ii) the privacy loss under rewarded data sharing. The type of data they share plays a more minor role, though a positive one for privacy preservation. The models align well with the perception of individuals: 29.4, 37.85, and 32.75% for sensor type, collector, and context, respectively (Fig. 6b). In contrast, for data-sharing choices of individuals with reward gains, the dominant criterion is the type of sensor data with a 45.4% of relative importance over the data collector and context with 24.55 and 30.01%, respectively. The collectors and contexts explain loss of rewards, while the type of sensor, and in particular the GPS, explains reward gain. GPS, as a privacy-sensitive sensor, provides high gain of rewards, and individuals are likely to be accustomed with apps accessing their GPS data, which, in turn, is likely to reduce privacy preservation. Choices that improve rewards suggest a radically different decision frame than the ones that improve privacy: *a shift from protecting to sharing GPS data without strongly distinguishing anymore the data collectors and contexts*.

Figure 6b also provides the following observations: The relative importance of the perceived privacy sensitivity over the 12 data-sharing elements is positively correlated with all models based on privacy: $R=0.97,t(10)=12.22,p=2.46\times 10^{-7}$ for rewarded data sharing, R=0.84,t(10)=4.87,p=0.00066 for intrinsic-rewarded, R=0.69,t(10)=3.025,p=0.013 for the coordinated data sharing and R=0.67,t(10)=2.89,p=0.016 for the intrinsic one. All models come with a positive relative importance for GPS (12.67%), corporation (15.16%), and social networking (20.42%), while negative one for accelerometer (−11.85%), light (−8.9%), educational institutes (−21.52%), transportation (−6.13%), and health (−6.63%).

### From intrinsic to rewarded data sharing: five behavior changes

#### Identifying group behaviors

**Fig. 7.**
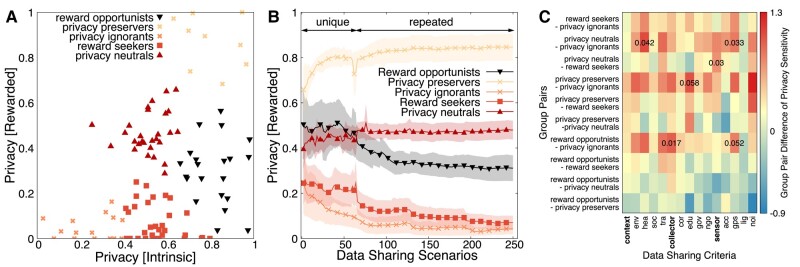
Five key group behaviors in data sharing and their effects. A) Data-sharing group behaviors for intrinsic vs. rewarded data sharing. B) Privacy of groups over consecutive rewarded data-sharing choices. C) Group pair differences of privacy sensitivity over data-sharing criteria.

Table 1 provides an exemplary of all nine possible behavioral transitions that can happen in data sharing as a result of introducing monetary rewards. A clustering and stability analysis are performed in the experimental data projected in Fig. 7a (intrinsic vs. first rewarded), which reveal five robust behavioral patterns out of the nine possible ones (similar groups are observed for intrinsic vs. second rewarded). See the Extraction and validation of group behavior section for more information. Some individuals are oblivious to rewards. Yet, these are the ones who intrinsically share a significant amount of data (*privacy ignorants* and *privacy neutrals*) or do not share data (*privacy preservers*). *Reward seekers* increase the data-sharing level when rewarded, while *reward opportunists* intrinsically preserve privacy but eventually share a significant amount data when rewarded. It is astonishing that a moderate sacrifice of privacy preservation by rewards is not observed (privacy sacrificers in Table 1), meaning that rewards significantly polarize individuals to keep protecting privacy or give up significant privacy. There are also no cases observed in which rewards motivate change to privacy protection; however, rewards reinforce privacy protection for privacy preservers.

**Table 1.** Exemplary of possible group behaviors with and without rewards in data sharing.

	Without rewards			With rewards		
Data sharing:	Low	Moderate	High	Low	Moderate	High
Privacy ignorants			✓			✓
Privacy neutrals		✓			✓	
Privacy preservers	✓			✓		
Rewards seekers		✓				✓
Rewards opportunists	✓					✓
Privacy sacrificers	✗				✗	
Reward opposers (sharer)			✗	✗		
Reward opposers (neutral)		✗		✗		
Reward sacrificer (sharer)			✗		✗	

A low, moderate, and high level of data sharing is assumed for illustration purposes. ✓: denotes the observed group behaviors. ✗: denotes the unobserved group behaviors.

#### Groups behavior converges to stable, while boundary ones polarize

The behavioral pattern of privacy sacrificer (Table 1) is found to be a transient one and observed within the reward opportunists during the first unique responses to the 64 data-sharing scenarios (see Fig. 7b). When though these individuals get more involved in reevaluating their decisions, they converge to a further privacy sacrifice of 30.9%. The minimum number of questions answered by all groups is 250. This incremental privacy decline in reoccurring decision-making is also observed in reward seekers and privacy ignorants that decrease their privacy level by 55.7 and 64.8%, respectively. On the contrary, privacy preservers show a further increase in their privacy by 8.7% as they reevaluate their data-sharing decisions. Such a privacy increase of 8.1% is also observed for privacy neutrals.

Strikingly, the two boundary behavioral patterns of privacy preservers and privacy ignorants show polarization from the very first data-sharing decisions. These individuals reinforce the privacy preservation and privacy ignorance respectively throughout the choices they make and regardless of whether these choices are the primary ones (the first 64 questions) or the reassessments (the follow-up reinvoked questions). A similar behavior is documented for data sharing in social media (8, 15, 44), though this is the first evidence of such behavior in a broader context, involving both privacy and rewards dilemmas.

#### How privacy sensitivity of data-sharing criteria explains group behaviors

Figure 7c shows all group pairs and the differences between these groups in terms of how privacy sensitive they regard each data-sharing criterion (attitudinal). Statistically significant observations (p≤0.05) and those close to the significance threshold are marked in Fig. 7c. These results are derived with a post hoc Tukey’s range test (α=0.05) after a one-way ANOVA. The independent variable is calculated within the groups by the privacy change from intrinsic to rewarded data sharing. The dependent variables are the privacy sensitivity of the data-sharing criteria and their elements. Several of these criteria explain the data-sharing groups with a statistical significance (see Fig. S16, Section S13): transportation (F(4,111)=2.779,p=0.03), data collector (F(4,110)=2.463,p=0.027), sensor (F(4,110)=  2.686,p=0.031), GPS (F(4,110)=2.201,p=0.033), and noise (F(4,110)=3.573,p=0.056).

In Fig. 7c, the data collector (p=0.017) and the GPS sensor (p=0.052) explain the privacy-sensitivity difference between reward opportunists and privacy ignorants: rewarded individuals of these groups share a significant level of data, while reward opportunists preserve privacy without rewards. Compared to privacy ignorants, reward opportunists find data collector and GPS more privacy intrusive by 24.2 and 20.4%. Similarly, the context of health (p=0.042) and the GPS sensor (p=0.033) explain the divergence between privacy neutrals and privacy ignorants. Privacy neutrals find these two data-sharing criteria 26.6 and 20.9% more privacy intrusive than privacy ignorants. Privacy neutrals also find sensors (p=0.033) more privacy intrusive than reward seekers by 18%, which explains the higher data sharing of rewards seekers under rewards. Finally, the data-sharing criterion of educational institute determines when individuals share a very high or very low level of data with or without rewards: privacy preservers find the context of education (p=0.058) 25.9% more privacy intrusive than privacy ignorants.

## Discussion

The findings reveal that a significant privacy recovery is attainable within the modus operandi of a data collective. This is a radical shift from mainstream thought of privacy as a personal value to privacy as a collective value (45), a public good shared within a community of citizens generating data. Coordinated data sharing supported by a trustworthy decentralized AI automates and scales up collective arrangements for sharing under the doctrine “as little as possible as much as necessary.” Such optimized arrangements would be otherwise too complex and expensive to achieve in a transparent way with existing top-down privacy policies and regulations or even with automated data-access committees (46).

Findings also reveal that data collectives create tangible benefits for online service providers that collect or access data shared in a coordinated way: data-collection costs drop down dramatically, and data are used more purposefully to deliver the required quality of service. This can create further remarkable cost reductions such as reduced data storage, security, energy, and carbon footprint costs as well as costs for solving legal disputes that are more likely to incur when dealing with excessive personal data.

Within rising information asymmetries and monopolies of knowledge in existing data markets and big tech, the capability of data collectives to coordinate data sharing at large-scale has been so far a gap (47, 48). This is underlined in promising solutions from political and economic theory such as data-owning democracy (49), digital socialism (47), and peer-to-peer digital commons (50). Establishing data collectives at a community or municipality level can create alternative forms of data ownership and control; they can empower citizens participation based on an agenda of using digital assets for priorities such as social welfare and environmental sustainability (48, 51). These blueprints can be the basis of alternative data-market designs that encourage business models based on social innovation without over-relying on excessive free personal data. Data collectives can further benefit from scale, for instance, increasing individuals who coordinate their data-sharing decisions or increasing individuals’ flexibility contributions by generating more alternative data-sharing options. The AI system based on collective learning has a higher degree of freedom to calculate data-sharing choices that match the required data and recover more privacy in larger populations (25). It is also decentralized to make coordination more resilient to computational bottlenecks (25, 52).

Science can also benefit from data collectives. They can scale up open data and citizen science initiatives, while improving the transparency and reproducibility of research. Moreover, data collectives can be a response to the current opaque models of generative AI such as ChatGPT. Selective data shared as a result of coordination can be used to train open and more transparent generative AI models, ethically aligned to community values. This could be a new type of “curricula” for training AI, institutionalized in a bottom-up way via data collectives.

Choices under intrinsic and rewarded data sharing prioritize different criteria. Individuals better distinguish data collectors and contexts than the type of data they share. In contrast, rewarded individuals that give up privacy better distinguish the type of data they share, and in particular the GPS. Thus, rewards diminish the importance of who collects data and for what purpose. In this case, data collectors may have no competitive advantage against each other but instead excessive and irrelevant data that increase their costs and risks.

The perceived privacy sensitivity of the data-sharing criteria explains different key data-sharing behaviors (groups), for instance, individuals who do not preserve privacy vs. individuals who sacrifice privacy under rewards. Raising awareness about the privacy sensitivity of data collectors can influence data-sharing decisions. This has implications for how privacy policies and data consents are designed to be more transparent and user-friendly. Data-sharing choices that preserve and give up significant privacy tend to polarize, thus highlighting the value of privacy for individuals who have it rather than for the ones who do not (15). Coordinated data sharing breaks this vicious cycle by redistributing the privacy cost within the individuals for the benefit of all. This demonstrates opportunities for digitally networked societies without borders to reconcile different cultural norms on privacy.

Future work can unleash further opportunities to reclaim privacy in the digital age: Spatiotemporal coordinated data sharing can automate and scale up the “*right to be forgotten*,” which improves both privacy control and the willingness to share data, e.g. 10–18% (13). The feasibility of collective learning using optimization scenarios in time and space are earlier demonstrated for Smart City applications (25). Nevertheless, defining and conveying to individuals the context of data use is not always straightforward and further work is required in this area, for instance, semantics and ontologies (46). Moreover, beyond purposeful data sharing, speculative data analysis out of a specific context can also encourage innovation and creativity. In such scenarios, data collectors may have a more significant role for trust in data-sharing decisions. The acceptance of coordinated data-sharing recommendations requires a follow-up study, in particular, the incentives and the interface design of the AI system for the broader population. Notwithstanding, results show that coordinated data sharing comes with lower levels of shared data compared to rewarded data sharing, therefore, this is itself a significant incentive for individuals to use and trust the proposed solution. Moreover, earlier results demonstrate significant coordination capacity even when large portions of the population are not flexible (53). The explainability of coordinated data sharing based on decentralized AI is particularly challenging and is expected to further shield the trust on data collectives.

## Methods

We outline here the experimental design and the developed technical infrastructure. We also illustrate the methods with which we analyzed the experimental data and the AI-based decision-support system with which coordinated data sharing is performed.

### Living-lab experimental design

**Fig. 8.**
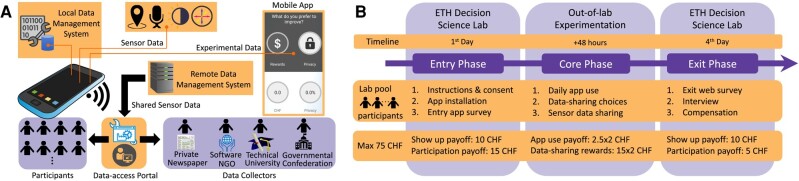
A data-collection infrastructure used for the design of a novel “living-lab” experiment of high realism and rigor. A) Data are collected via smartphones and are made accessible to data collectors according to the privileges given by participants. B) The experiment consists of three phases in and out of the lab.

A novel design for a “living-lab” experiment is introduced. It defines a *mixed-mode* experiment that seamlessly integrates in participants’ everyday life, while the overall experimental process is orchestrated via the controlled environment and experimental protocols of the Decision Science Laboratory (DeSciL) of ETH Zurich (54). The proposed experiment has received ethical approval by DeSciL and the Ethics Commission at ETH Zurich (#EK 2016-N-40). Informed consent is obtained from all participants. To improve the realism of the experiment and comply to the nondeceiving policy of DeSciL, letters of support were collected from data collectors to confirm their interest in accessing the collected sensor data of participants. The study consist of three phases: (i) *entry*, (ii) *core*, and (iii) *exit*. Fig. 8 provides an outline of the overall experimental process and the developed data-collection infrastructure (details are documented in Section S3).

#### Recruitment approach and sampling biases

The living-lab experimentation involves the recruitment of 123 participants during the entry phase, out of which 116 completed the exit phase and 89 participated in all phases. Aggregated privacy-reward records for all experimental conditions is found for 84 participants. Responses to the data-sharing scenarios for all experimental conditions are found for 73 participants. In the context of this study, a higher number of participants is particularly challenging and probably unrealistic as it requires significantly more resources for compensation/infrastructure, sacrifice of rigor, and much looser control of the experimental process. Instead, priority is given to a satisfactory compensation per participant for active participation in all experimental phases (see Section S3.5) and by incentivizing appropriately a large number of data-sharing choices: 27,403 in total. Moreover, the development of a data-collection platform, including the data-access web portal and the mixed-mode experimental process, preserves an eminent realism, yet in well-controlled laboratory conditions that result at the end in a novel high-quality dataset to perform causal inference.

Participants were recruited from the DeSciL pool (55), mainly consisting of students of ETH Zurich and University of Zurich (see the invitation in Section S2.2). This pool is not representative of the population and is subject to sampling biases. However, smartphone users, who use a broad range of apps that require sharing of sensor data are mainly young people (56–58), and therefore the students’ profile fits well with the nature of the conducted experiment. Participants with technological literacy are also more likely to be familiar with data-sharing dilemmas involving a privacy cost to gain access to smartphone app services. Studying such a sample of participants can make results more compelling as shown in earlier experiments conducted on such recruitment basis (59). Only Android smartphone users are recruited, who are a large portion of the population, for instance, 39.8% in Switzerland, 68.6% in Europe, and 72% worldwide in 2016 according to StatCounter. Moreover, several smartphone apps with data-sharing decisions are made for both Android and iOS. Therefore, there is no substantial evidence to suggest different decision patterns among the market share in the population as also supported in earlier work (59). Recruitment is performed in eight sessions on a weekly basis. To eliminate any further temporal bias, each of the three phases in Fig. 8 took place on the same day of the week. Table S2 provides an overview of the experimental sessions.

#### Entry phase

It takes place at DeSciL and it involves the following: (i) Collection of basic demographics about participants and information about their privacy profile using the survey questions of Table S4. (ii) Use of the privacy-intrusion level assigned to each data-sharing criterion and its elements (Questions B.9–B.12) to calculate the attitudinal data sharing and to calibrate the calculation of the monetary rewards for the core phase according to the model illustrated in Section S1. (iii) Collection of the intrinsic data-sharing decisions by letting participants choose once the data-sharing level for each of the 64 data-sharing scenarios (see Fig. S3b). The following question implements the data-sharing scenarios:

Factorial Question Please choose the amount of <sensortype> sensor data shared with <datacollector> to be used in the context of <context>.

There are in total five possible data-sharing levels to choose from (see Fig. S3b).

#### Core phase

**Fig. 9.**
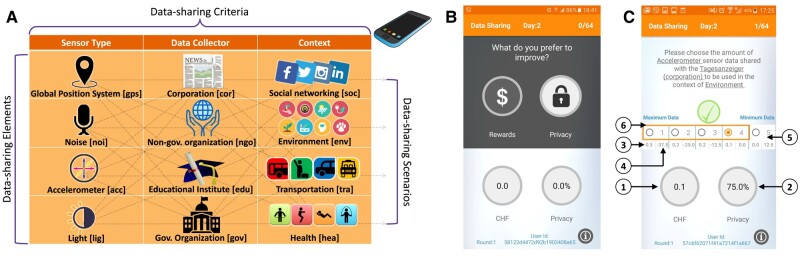
The studied 4×4×4 full factorial design for smartphone data sharing and the key experimental functionality of the smartphone app. A) It consists of three data-sharing criteria, each with 4 elements creating 64 combinations of data-sharing scenarios. Each scenario involves a choice of what data to share, to which data collector and for what purpose. The choice of the exact sensors, collectors and contexts is outlined in Section S3.2. The labels in the brackets are used in the plots of this article. B) Privacy vs. rewards dilemma. C) Data-sharing scenario choice. Arrows on the app screen: (1) Accumulated rewards. (2) Privacy level, e.g. 75% corresponds to sensor data sampling every 120 s as explained to participants, see Fig. S1. (3) Gain/loss of rewards for a particular option. (4) Gain/loss of privacy for a certain option. (5) Data-sharing options. (6) Options in the improvement box.

It takes place out of the lab and lasts for 2 days (48 h), starting right after the completion of the entry phase. During the 24 h of each day, participants are voluntarily involved in an (unlimited) sequence of dilemmas of either improving their privacy or rewards by sharing less or more data respectively in a data-sharing scenario. Figure 9 illustrates the two app screens for the privacy-rewards dilemma and the data-sharing scenario that follows. First, participants decide what to improve based on their privacy-rewards balance they currently have (Fig. 9b). Next, a data-sharing scenario is automatically retrieved with the latest choice made (Fig. 9c), marking the options that fulfill their goal (the improvement box, see Arrow 6). The retrieved scenario is the one that maximizes the improvement of the chosen goal, i.e. privacy or rewards. For each option, the app informs participants about the rewards and privacy they gain or lose (Arrows 3 and 4, respectively). After a choice, the participant moves back to the main screen of Fig. 9b with an updated privacy-rewards balance.

The first unique 64 data-sharing scenarios are the ones that participants have decided about during the entry phase. The difference in this core phase is that data sharing is rewarded based on two factors defined in the data-sharing model (see Section S1): (i) the data-sharing level (the higher, the more rewards) and (ii) how privacy-intrusive the data-sharing scenario is according to each participant. More rewards are allocated to data-sharing scenarios involving criteria regarded highly privacy intrusive by a participant. The latter personalization is derived from the responses of the entry phase (Questions B.9–B.12 in Table S4) without explicitly making participants aware of this.

Within the 24 h, participants can change their goal based on their privacy-reward balance. They continue responding to further retrieved data-sharing scenarios that can satisfy their goal, i.e. improve privacy or rewards, see Fig. 9b. This allows studying how data-sharing decisions evolve. Each decision in a data-sharing scenario overwrites the previous one for the calculation of the privacy-reward balance. At the end of the 24 h, the process completes by locking the decisions of the 64 scenarios and sharing the data to the data-access web portal. This process runs for 2 days to validate the results, confirming similar data-sharing behavior at both days (see Figs. 3a and S9a and b).

#### Exit phase

The participants of each experimental session return to DeSciL on the fourth day. They answer a survey questionnaire, participate in an interview and receive their calculated compensation. The survey consists of questions that cover the following aspects (see Tables S6 to S9): (i) smartphone use, (ii) user interface and functionality of the app, (iii) rewards and privacy, and (iv) experimental process. The data collected during this phase have a supportive role serving the validation and interpretation of the results produced during the entry and core phase. See Section S3.4 for further details.

#### Compensation and monetary incentives

Participants are compensated for their engagement in the experiment as well as for the sensor data they share. The engagement covers (i) showing up in the lab (2×10=20 CHF), (ii) completing the lab activities (15+5=20 CHF) and (iii) using the app in terms of answering at least once all 64 data-sharing scenarios (2×2.5=5 CHF). The rewards for the app use is distributed with a geomentric progression over the data-sharing scenarios to eliminate dropout effects (see Section S3.5). Those who successfully complete all experimental phases receive the total fixed compensation of 45 CHF and an additional maximum reward of 2×15=30 CHF based on the amount of shared data. Figure 8b shows how the total maximum amount of 75 CHF is allocated over the experimental process. Section S3.5 further motivates the allocation of these compensations.

### Technical infrastructure

Figure 8a outlines the technical infrastructure developed to serve the designed experimental process. Two types of data are collected by the smartphone app: (i) the sensor data that participants explicitly choose to share and (ii) all data from participants’ choices and survey answers used for the analysis. These data are stored on a remote server and locally on the smartphone for redundancy so that they can be restored during the exit phase by moderators in case of software or communication failures.

The developed infrastructure consists of the following interactive systems: (i) the *local*, (ii) *remote data-management system*, (iii) the *smartphone app*, and (iv) the *data-access web portal*. The two data-management systems synchronize and secure the shared sensor data as well as the experimental data. The smartphone app is developed to run on Android devices. The data-access web portal stores the shared data and provides authorized access to the registered participants of the experiment as well as the data collectors involved in the data-sharing scenarios. Making available this system improves the realism of the experiment by realizing the actual data-sharing decisions, while allowing the experimental design to comply with the nondeceiving policy of DeSciL. See Section S4 for further details.

### Privacy calculations for sensors, collectors, and contexts

The privacy measurements in Fig. 4a are made as follows: In the case of the attitudinal data-sharing condition, the mean privacy level is calculated by normalizing (in [0,1] over all participants) the privacy sensitivity reported in the Questions B.10–B.12 during the entry phase. In the intrinsic, rewarded and coordinated data-sharing conditions, the privacy level of a certain sensor, data collector or context is the normalized privacy mean across all participants for 16/64 data-sharing scenarios that contain this respectively (see Fig. 3a). In the coordinated data-sharing conditions, this is calculated using the mean privacy level of the data-sharing scenarios selected over all 10 repetitions of the coordination with a random permutation in the positioning of the agents (see the Coordinated data-sharing via decentralized AI section for more information).

The expected privacy level of a data-sharing scenario (see shaded areas in Fig. 3a) is calculated by the mean privacy level of the sensor, collector, and context that comprise the data-sharing scenario. The expected privacy level of a certain sensor, data collector, or context is the mean expected privacy level over 16/64 data-sharing scenarios containing this. The relative difference between the actual privacy level and the expected one defines the *privacy reinforcement*. Detailed measurements are illustrated in Fig. S13, Section S10.

### Coordinated data-sharing via decentralized AI

Coordinated data sharing is modeled as a decentralized discrete-choice multiagent combinatorial optimization problem. It is designed to recover excessive privacy loss of the rewarded data sharing. A decision-support system implements the optimization that achieves the coordination. The discrete choice model and the coordination method are outlined below.

#### Data-sharing plans and elicitation of privacy sensitivity

Each participant comes with three data-sharing plans extracted from the living-lab experiment as follows: each plan is a sequence of 64 real values that represent the data-sharing choices made at each scenario and each experimental condition: intrinsic, first rewarded and second rewarded. Each plan has a privacy cost represented by a real value. It is calculated by the mean normalized level (in [0,1]) of shared data over the data-sharing scenarios. Alternative privacy valuation schemes are assessed in Section S9.

#### Steering data sharing using privacy-preservation goal signals

A goal signal represents a data-collection scenario with the minimum required data to enable a data-driven service or application (32–34). Five privacy-preservation goal signals for data sharing are generated using the intrinsic data-sharing choices of participants. Each goal signal is a sequence of 64 values corresponding to the data-sharing scenarios. For each data-sharing option out of the five possible ones, a goal signal is calculated with the 64 values representing the probability of participants choosing this data-sharing option without rewards. Similarly with the data-sharing options, the five goal signals are referred to within the range of very low to very high privacy preservation. Figure S10, Section S7 illustrates the five goal signals.

#### Coordinated data sharing

The goal of the data collective is to choose and aggregate (sum up element wise) the data-sharing plans of all individuals such that the resulting signal matches a given goal signal. This matching is measured here with the residual sum of squares between these two signals (standardized). As this goal cannot be satisfied by letting individual participants choosing independently the plan with the best matching (minimizing a nonlinear cost function), coordination between participants’ choices is required. This discrete-choice coordination problem is combinatorial NP-hard and requires approximating solutions (25). The coordination capability can be generalized to a multiobjective combinatorial optimization problem in which the data collective minimizes the following cost function:


(1) $$\begin{array}{rl} (1-\alpha -\beta )\;\times & \text{ privacy inefficiency}+\alpha \times \text{ privacy unfairness}\\ & +\beta \times \text{ privacy cost}, \end{array}$$

where privacy inefficiency is the data sharing mismatch measured by the residual sum of squares between the aggregated data-sharing plans and the goal signal. The privacy cost is the mean cost of the selected plans and the privacy unfairness is the dispersion (variance) of privacy cost over individuals. The parameters *α* and *β*, for α+β=1 and α,β∈[0,1], are self-determined by each individual and model a behavioral continuum between selfish vs. altruistic behavior in terms of data sharing. A selfish individual that minimizes privacy cost without coordinating its data sharing with other individuals is determined by β=1,α=0. An individual that minimizes the collective privacy inefficiency without counting its personal privacy cost is an altruistic one by β=0,α=0. These altruistic individuals can balance for privacy unfairness by increasing the *α* parameter.

#### A decentralized computational approach for coordination

The collective-learning method of I-EPOS is used to cope with the computational and communication complexity of the coordinated data-sharing problem (25). This algorithm is used as a decision-support system that automates and scales up the coordination, which would otherwise be too complex and infeasible for humans to perform without digital assistance. As featured by UNESCO IRCAI (29), this method is particularly fitting in this privacy context: (i) The algorithm itself is privacy-preserving by design as it exclusively relies on exchanging aggregated data sharing choices rather than individual ones. The use of differential privacy and homomorphic encryption can also enhance the overall security of information aggregation, which is an additional privacy protection on top of the privacy recovery illustrated in this article. (ii) The algorithm is highly cost-effective with a low computational and communication complexity compared to other multiagent approaches for combinatorial optimization problems (25). The data-sharing choices calculated by the algorithm can rapidly match the goal signal with a low communication exchange between the agents. (iii) The algorithm is open-source, decentralized, and can scale up without relying on a trusted third party, which makes it particularly applicable for bottom-up data collectives. (iv) The algorithm can operate in different faulty environments and application scenarios (52).

#### Collective-learning parameterization

Agents are self-organized in a binary balanced tree within which they are positioned randomly. Coordination repeats 10 times, each with a different random positioning of the agents. For each random positioning, collective learning runs for 50 learning iterations. Each iteration proceeds from leaves to root and back to leaves. It results in the selection of data-sharing plans that minimize at an aggregate level the cost function in Eq. 1. More information about the algorithm can be found in earlier work (25).

### Causal inference with conjoint analysis

The complete factorial design of three data-sharing criteria each with four elements results in 64 scenarios encoded by a sequence of 12−3=9 dummy variables. These represent the membership of a certain sensor, collector, and context in a data-sharing scenario. Multiple linear regression models are constructed using as independent variables the nine dummy variables (4−1=3 variables per data-sharing element are used to resolve the linear dependency problem in multiple regression). The dependent variables that distinguish the regression models include the following (Fig. 6): privacy (intrinsic, intrinsic-second rewarded, coordinated with the very low privacy-preservation goal) and gained rewards (first and second rewarded data sharing with those individuals who intend and do improve rewards as in Fig. 9). These privacy and reward values across the 64 data-sharing scenarios of the full factorial design are used for a rating-based conjoint analysis. Other regression models with lower statistical power are assessed and further illustrated in Fig. S14, Table S13, and Section S11.

The regression models result in the 12 coefficients for each data-sharing element as shown in Fig. 6a. Together with a constant (Table S13), they predict the depend variable. Using the coefficients, the partworth utilities are estimated that calculate the relative importance of each data-sharing criterion and element (Eqs. S11 and S12). For each data-sharing element, the relative importance is calculated across the elements of the criterion it belongs (Eq. S12) or across all elements (Eq. S13). The latter is shown in Fig. S15. The conjoint analysis models are compared to the mean relative perceived privacy sensitivity as declared by participants in the Questions B.9–B.12 in Table S4.

### Extraction and validation of group behavior

#### How groups are extracted

To extract the data-sharing group behaviors, the participants’ privacy level under intrinsic and first/second rewarded data sharing are clustered using three clustering techniques of R: (i) k-means (60) (kmeans), (ii) hierarchical clustering (61, 62) (hclust), and (iii) partitioning around medoids (63) (pamkCBI). A subset of 110 participants were clustered that made both intrinsic and rewarded data-sharing decisions. An optimum number of five clusters is confirmed in all three methods that correspond to the data-sharing groups marked in Fig. 7a. An exemplary of observed and unobserved group behaviors is outlined in Table 1.

#### How groups are validated

In the case of k-means and hierarchical clustering, the optimum number of five clusters is derived by performing a bootstrap evaluation (clusterboot of R) of the clusters (64). It assesses both the stability of the clusters and the stability of different clustering algorithms. The pamkCBI algorithm performs partitioning around medoids. The number of clusters is estimated by the optimum average silhouette width (65, 66). However, a bootstrap evaluation is also performed for pamkCBI for a complete comparison of the three algorithms. An outline of the clusters stability (mean Jaccard similarity) and the number of dissolved clusters for 100 bootstrap iterations is given in Table S14. Visual inspections show that all three algorithms find the same clusters, while k-means achieves a mean Jaccard similarity (bootmean) higher than 0.75 for all clusters, which indicates stable clusters. As such, the groups of k-means are analyzed in this article (Fig. 7). Note also that the population split over the data-sharing groups matches well to Westin’s general population privacy indexes, see further Section S12.

## Supplementary Material

pgae029_Supplementary_Data

## Data Availability

The collected data of the living-lab experiment are made available at: https://doi.org/10.6084/m9.figshare.21750158. The generated plans are made part of the following planning portfolio: https://doi.org/10.6084/m9.figshare.7806548.v5. The source code of the AI system is under active development at https://github.com/epournaras/epos. Source code used and developed for this article is made available at https://doi.org/10.5281/zenodo.7457575.

## Preprints

This manuscript was posted on a preprint: https://doi.org/10.48550/arXiv.2301.05995.
