# Supplementary material for: Collective privacy recovery: Data-sharing coordination via decentralized artificial intelligence
Source: PNAS Nexus. 2024 Jan 22;3(2):pgae029. doi: 10.1093/pnasnexus/pgae029 (PMC10847902; doi:10.1093/pnasnexus/pgae029)
Supplement: pgae029_Supplementary_Data [file pgae029_supplementary_data.pdf]

Collective Privacy Recovery: Data-sharing Coordination via Decentralized Artificial Intelligence

Supplementary Information

Evangelos Pournaras[1], Mark Christopher Ballandies[2], Stefano Bennati[2] and Chien-fei Chen[3]

[1]School of Computing, University of Leeds, Leeds, UK, E-mail: e.pournaras@leeds.ac.uk
[2]Computational Social Science, ETH Zurich, Zurich, Switzerland, E-mails: mark.ballandies@ethz.ch, stefano@bennati.me
[3]Institute for a Secure and Sustainable Environment, University of Tennessee, Knoxville, E-mail: cchen26@utk.edu

January 14, 2024

Contents

1 General Data-Sharing Model **2**
1.1 Data-sharing criteria . . . 2
1.2 A weighting scheme for personalized privacy valuation . . . 3
1.3 Calculating rewards and privacy . . . 4

2 Recruitment Process **4**
2.1 Recruitment sessions . . . 4
2.2 E-mail invitation for recruitment . . . 5

3 Experimental Design **6**
3.1 Preparatory phase . . . 6
3.2 Entry phase . . . 6
3.3 Core phase . . . 12
3.4 Exit phase . . . 13
3.5 Compensation and monetary incentives . . . 16

4 Implementation of the Technical Infrastructure **17**

5 The Privacy and Rewards Gain of Data-sharing Scenarios **17**

[1]Corresponding author: Evangelos Pournaras, School of Computing, University of Leeds, Leeds, UK, E-mail: e.pournaras@leeds.ac.uk

6 Privacy Loss and Rewarded Data-sharing Choices of Groups **17**

7 Goal Signals for Coordinated Data Sharing **17**

8 Data-sharing Mismatch **18**

9 Valuations of Collective Privacy Recovery **18**

10 Privacy Reinforcement **22**

11 Conjoint Analysis **22**

12 Validation of Groups **26**

13 Analysis of Variance for Data-sharing Criteria and Groups **27**

1 General Data-Sharing Model

This section provides the mathematical formulation of human data-sharing choices under personalized (monetary) incentives. Table S1 provides an overview of the mathematical notations.

1.1 Data-sharing criteria

Let k factors, referred to as *criteria*, govern the level of data sharing that an individual, i.e. a *citizen*, chooses. This ranges from sharing no data to sharing all locally available data in an individual's device such as a smartphone. Each criterion $u \in \{1, ..., k\}$ has a number of possible *elements* l_u. For instance, the type of sensor data is a criterion with the following elements (see Figure 9a in the main paper): GPS location, light sensor, etc. The former element may be regarded more privacy intrusive than the latter one. The total number:

$$m = \prod_{u=1}^{k} l_u, \tag{1}$$

of combinations between the l_u elements of the k criteria define the *scenarios* of data sharing, which are the ones studied in this paper. For each data-sharing scenario $j \in \{1, ..., m\}$, individuals have a number of z discrete *data-sharing options*, where the first option corresponds to sharing all collected data, whereas the zth option corresponds to sharing no data. Each individual i selects a *data-sharing level* $s_{i,j} \in \{1, ..., z\}$ for scenario j. For simplicity, assume that the actual level of data sharing decreases linearly from 1 to z by, for instance, averaging, obfuscating or resampling the data to share (e.g. with a period proportional to $s_{i,j}$). The data-sharing level $s_{i,j}$ is a result of a function:

$$s_{i,j} = f_i(D_j), \tag{2}$$

where $D_j = (d_{j,u})_{u=1}^{k}$ represents the *data-sharing scenario* j as the sequence of elements $d_{j,u} \in \{1, ..., l_u\}$ over all k criteria. For the sake of simplicity in the model illustration, the number of criteria k and the number of elements l_u for each criterion u are assumed finite and fixed for all n individuals.

Table S1: An overview of the mathematical symbols.

Symbol	Interpretation
k	Number of data-sharing criteria
u	A data-sharing criterion
l_u	Number of elements of a criterion u
m	Number of data-sharing scenarios
i	An individual
j	A data-sharing scenario index
z	Number of data-sharing levels
$s_{i,j}$	The selected data-sharing level of individual i for a data-sharing scenario j
D_j	A data-sharing scenario
$f_i(D_j)$	A data-sharing decision function of individual i in a data-sharing scenario D_j
$d_{j,u}$	An element of criterion u in a data-sharing scenario j
n	Number of individuals
$w_{i,u}$	The weight of criterion u by an individual i
o	The index of an element of a data-sharing criterion
$w_{i,o,u}$	The weight of an element o of a criterion u by an individual i
$W_{i,j}$	The weight of a data-sharing scenario j by an individual i
B	Maximum (monetary) budget
B_{p}	Rewards for participation
B_{s}	Rewards for data sharing
$\hat{r}_{i,j}$	The maximum rewards of individual i for a data-sharing scenario j
W_i	The total weight of all data-sharing scenarios by an individual i
$r_{i,j}$	The actual rewards of an individual i for a data-sharing scenario j
p_i	The privacy level of an individual i derived from the data-sharing choices
$\lambda_{u,o}$	The coefficient of a data-sharing element o in the criterion u
$\mathcal{D}_{u,o}$	The dummy variable for the absence or presence of the data-sharing element o in the criterion u
ϵ	The error of the regression model
$\mathcal{P}_u$	The partworth utility (relative importance) of criterion u
$\hat{\mathcal{P}}_{u,o}$	The partworth utility (relative importance) of element o in criterion u among all criteria
$\mathcal{P}_{u,o}$	The partworth utility (relative importance) of element o within criterion u
P_j	The mean privacy level of a data-sharing scenario j
ε	The mismatch (absolute error) of data sharing from a privacy-preservation goal signal
R_j	The mean rewards level of a data-sharing scenario j
r_i	The rewards of individual i gained over the data-sharing scenarios
$\tilde{r}_i$	The hypothetical rewards of an individual i gained over the data-sharing scenarios under intrinsic data sharing
$C_i(r_i)$	The privacy cost of a data-sharing plan generated by individual i as a function of r_i
α, β	The weights of privacy unfairness and privacy cost respectively in the optimization cost function

1.2 A weighting scheme for personalized privacy valuation

Let the weight $w_{i,u} \in [0,1]$ denote how privacy-sensitive a criterion u is for an individual i relative to the rest of the criteria, such that $\sum_{u=1}^{k} w_{i,u} = 1$. Similarly, the weight $w_{i,o,u} \in [0,1]$ denotes how privacy-sensitive an individual i finds the element $o \in \{1,...,l_u\}$ of criterion u relative to the rest of the elements, such that $\sum_{o=1}^{l_u} w_{i,o,u} = 1$.

The weight $W_{i,j}$ of a data-sharing scenario j is determined by each criterion weight $w_{i,u}$ and each element weight $w_{i,o,u}$ it consists of as follows:

$$W_{i,j} = \sum_{u=1}^{k} w_{i,u} \cdot w_{i,o,u}, \tag{3}$$

where $o = d_{j,u}$ is the element of criterion u in the data-sharing scenario j.

The weighting scheme is used to model the heterogeneity in the availability of data that stems from the individuals' privacy perception, i.e. it is expected that privacy-sensitive data are more scarce and as a result they are also expected to have higher value in data sharing.

1.3 Calculating rewards and privacy

The calculation of rewards and privacy relies on the weighting scheme for personalized privacy valuation (Section 1.2). Assume there is a maximum (monetary) budget B to incentivize data sharing that is split as follows:

$$B = B_{\mathsf{p}} + B_{\mathsf{s}}, \tag{4}$$

where B_{p} rewards participation, meaning the cognitive effort required for individuals to make choices for all data-sharing scenarios and B_{s} rewards the actual data sharing respectively. Moreover, assume that the weights of each criterion/element represent the actual intrinsic privacy concerns of individuals. The maximum *rewards* $\hat{r}_{i,j}$ of an individual i for each data-sharing scenario j are allocated according to the self-determined privacy-intrusion level of the data-sharing scenario as follows:

$$\hat{r}_{i,j} = \frac{W_{i,j}}{W_i \cdot B_{\mathsf{s}}}, \tag{5}$$

where the weight W_i sums up the weights of all scenarios as follows:

$$W_i = \sum_{j=1}^{m} W_{i,j}. \tag{6}$$

The actual received rewards of an individual i with a data-sharing level $s_{i,j}$ under a data-sharing scenario j are calculated as follows:

$$r_{i,j} = \frac{z - s_{i,j}}{z - 1} \cdot \hat{r}_{i,j}. \tag{7}$$

The *privacy* of an individual i over all selections made in the m data-sharing scenarios is calculated as follows:

$$p_i = \frac{1}{m} \sum_{j=1}^{m} \frac{s_{i,j} - 1}{z - 1}. \tag{8}$$

2 Recruitment Process

The split of the recruitment process into multiple sessions as well as the invitation for the recruitment are illustrated in this section.

2.1 Recruitment sessions

Splitting the recruitment of participants and the experiment into multiple sessions serves the following: (i) Guaranteeing enough time to recruit participants from the pool. (ii) Having a manageable number of participants to moderate during the experimental process. (iii) Scale up the number of participants incrementally so that potential failures do not influence the overall experiment. The entry phase takes place on Mondays, the core phase during Mondays-Wednesdays and the exit phase on Thursdays.

A 93.6% of the participants did not know about the experiment before participating (Question D.28 in Table S9).

Table S2: Recruitment during the 8 experimental sessions performed.

**Session:**	**1**	**2**	**3**	**4**	**5**	**6**	**7**	**8**
**Entry Phase**	3.10.2016	17.10.2016	31.10.2016	7.11.2016	14.11.2016	21.11.2016	28.11.2016	5.12.2016
**Core Phase**	3-5.10.2016	17-20.10.2016	31.10-2.11.2016	7-9.11.2016	14-16.11.2016	21-23.11.2016	28-30.11.2016	5-7.12.2016
**Exit Phase**	6.10.2016	21.10.2016	3.11.2016	10.11.2016	17.11.2016	24.11.2016	1.12.2016	8.12.2016
**Num. of Participants**	15	13	11	16	15	13	19	21
**Compensations** (CHF)	666.0	813.0	746.0	840.0	943.0	805.0	1259.0	1283.0

2.2 E-mail invitation for recruitment

The invitation sent to the DeSciL pool of participants for the recruitment is presented below:

Dear <firstname> <lastname>,

We would like to invite you to an upcoming experiment '<experiment name>'. The experiment will be carried out in English, so you should be fluent in English in order to register for this study.

The experiment requires your participation at the ETH Decision Science Laboratory at TWO different days and the use of your mobile phone (Android only) at other two days to answer some questions.

Your participation in the experiment will be maximally compensated as follows:

Session 1: CHF 25.-
Core phase on mobile phone: Up to CHF 35.-
Session 2: CHF 15.-
Total: Up to CHF 75.-

You MUST attend both lab sessions in order to receive your payment. Furthermore, the following criteria are a requirement:

1) have and use an Android mobile phone, version 4.4 and above
2) have mobile Internet connection
3) Keep your phone switched on and adequately charged throughout the experiment
4) officially register for the study
5) arrive on time for the experiment at both days
6) install and use a mobile application to answer some question at two days
7) fulfill all experimental criteria including specified language proficiency
8) provide photo identification.

```
The sessions are scheduled as follows:
<session list>

If you want to participate, you can register by clicking on the following link:
<link>

(If you cannot click on the link, copy it to the clipboard by selecting it,
right click and choosing "Copy",
and then paste it into the address line in your browser
by right clicking there and choosing "Paste".)

Kind regards
ETH Decision Science Laboratory (DeSciL)
http://www.descil.ethz.ch/contact/
```

3 Experimental Design

The preparatory, entry, core and exit phase of the conducted experiment are outlined here in more detail. The compensation and monetary incentives introduced to engage participants are also illustrated.

3.1 Preparatory phase

The preparatory phase has a supportive role in the overall experiment as participants are neither compensated nor selected rigorously. Participants of the preparatory phase are selected from the network of employees at ETH Zurich (convenience sampling). The findings of the preparatory phase are not conclusive and mainly serve the design of the following phases. Nevertheless, this phase was scaled up to approximately 200 participants within 3 months, starting on 19.05.2016.

The preparatory phase consists of a web survey implemented in Qualtrics [1] with the questions outlined in Table S3. The goal of the preparatory phase is to provide some first insights about the perception of privacy from the perspective of the three studied aspects: sensor type, data collector and context. Questions A.9-A.14 are designed for this purpose. Questions A.6-A.8 provide information about the smartphone usage profiles, whereas, Question A.15 scrutinizes the type of incentives that motivate participants to share mobile sensor data. Questions A.1-A.5 collect demographic information.

3.2 Entry phase

The participants of each experimental session are verified by the DeSciL staff members by presenting a personal identification document, i.e. a passport or student card, nevertheless, the actual identity of the participants remains anonymous to the researchers using the lab. Participants are not allowed to interact with each other during the experiment and any questions need to be addressed in private directly to the experiment moderators by moving to a next room. In this way, biases about how each participant perceives and understands the experimental process are eliminated. This process is communicated to the participants before the beginning of the experiment. Next, participants are seated in a room with instructions about the experiment (Figure S1) and the information consent (Figure S2) placed in front of them.

Table S3: Survey questions for the preparatory phase.

ID	Question	Type	Options
A.1	What is your gender?	multiple choice, one selection	female, male
A.2	Which year were you born?	multiple choice, one selection	81 [1920,2000]
A.3	In which country have you lived most of your life?	multiple choice, one selection	all countries
A.4	What is the highest level of education you have completed?	multiple choice, one selection	less than high school, high school, some college, bachelors degree, masters degree, PhD degree
A.5	Which of the following categories best describes your employment status?	multiple choice, multiple selections	employed full time, employed part time, unemployed (looking for work), unemployed (not looking for work), retired, student, disabled
A.6	Which types of apps do you usually have on your smartphone?	multiple choice, multiple selections	education, entertainment, finance, game, health & fitness, medical, music & audio, news, productivity, shopping, social networking, transportation, travel, utility, weather
A.7	How many times do you check your mobile phone during the day (e.g. check notifications/time, open apps, etc.)?	multiple choice, one selection	1-35, 36-70, 71-100, 101-135, 135+
A.8	How concerned are you about the privacy of your mobile sensor data?	ratio scale	5 [Not at all concerned,extremely concerned]
A.9	Which level of privacy intrusion would you assign to the following mobile sensors?	group of questions	12 questions
A.9.1	Accelerometer (it measures the changes of the velocity of the smartphone)	ratio scale	5 [very low,very high]
A.9.2	Gyroscope (it measures the rotation/twist of the smartphone)	ratio scale	5 [very low,very high]
A.9.3	GPS (it measures the geographical location of the smartphone)	ratio scale	5 [very low,very high]
A.9.4	Proximity Sensor (it measures the physical distance of the smartphone from your body)	ratio scale	5 [very low,very high]
A.9.5	Ambient Light Sensor (it measures the ambient light level)	ratio scale	5 [very low,very high]
A.9.6	Battery Sensor (it measures the battery level)	ratio scale	5 [very low,very high]
A.9.7	Microphone (it measures several sound features. e.g. level of sound frequencies)	ratio scale	5 [very low,very high]
A.9.8	Camera	ratio scale	5 [very low,very high]
A.9.9	Thermometer (it measures the temperature of the device)	ratio scale	5 [very low,very high]
A.9.10	Air Humidity Sensor (it measures the relative humidity in a range 0-100%)	ratio scale	5 [very low,very high]
A.9.11	Barometer (it measures the atmospheric pressure)	ratio scale	5 [very low,very high]
A.9.12	Bluetooth (it measures the proximity of the device with other devices)	ratio scale	5 [very low,very high]
A.10	How important for your privacy is the type of sensor from which you share data?	ratio scale	5 [not at all important,extremely important]
A.11	Which level of privacy intrusion would you assign to the following stakeholders if you had to share your mobile sensor data with them?	group of questions	12 questions
A.11.1	Corporations/companies	ratio scale	5 [very low,very high]
A.11.2	Non-profitable/non-governmental organizations	ratio scale	5 [very low,very high]
A.11.3	Educational institutes (Public)	ratio scale	5 [very low,very high]
A.11.4	Governments and governmental organizations	ratio scale	5 [very low,very high]
A.12	How important for your privacy is the stakeholder you share your mobile sensor data with?	ratio scale	5 [not at all important,extremely important]
A.13	Which level of privacy-intrusion would you assign to the following contexts of apps with access to your mobile sensor data?	group of questions	9 questions
A.13.1	Education	ratio scale	5 [very low,very high]
A.13.2	Entertainment	ratio scale	5 [very low,very high]
A.13.3	Environment	ratio scale	5 [very low,very high]
A.13.4	Finance	ratio scale	5 [very low,very high]
A.13.5	Health	ratio scale	5 [very low,very high]
A.13.6	Shopping	ratio scale	5 [very low,very high]
A.13.7	Social networking	ratio scale	5 [very low,very high]
A.13.8	Training	ratio scale	5 [very low,very high]
A.13.9	Transportation/Traveling	ratio scale	5 [very low,very high]
A.14	How important is for your privacy the context of apps in which you share your mobile sensor data?	ratio scale	5 [not at all important,extremely important]
A.15	Select one or more incentives which would motivate you to share your mobile sensor data	multiple choice, multiple selections	money, vouchers/discounts on services and stores, free access to additional services (maps, recommended apps, etc.), free access to data, contributing to public good, contributing data if my friends did, contributing data without incentives

The Android app was made available in Google Play online store for the participants to download, see Figure S3a. The app generates locally in the background a unique ID used as identifier of the

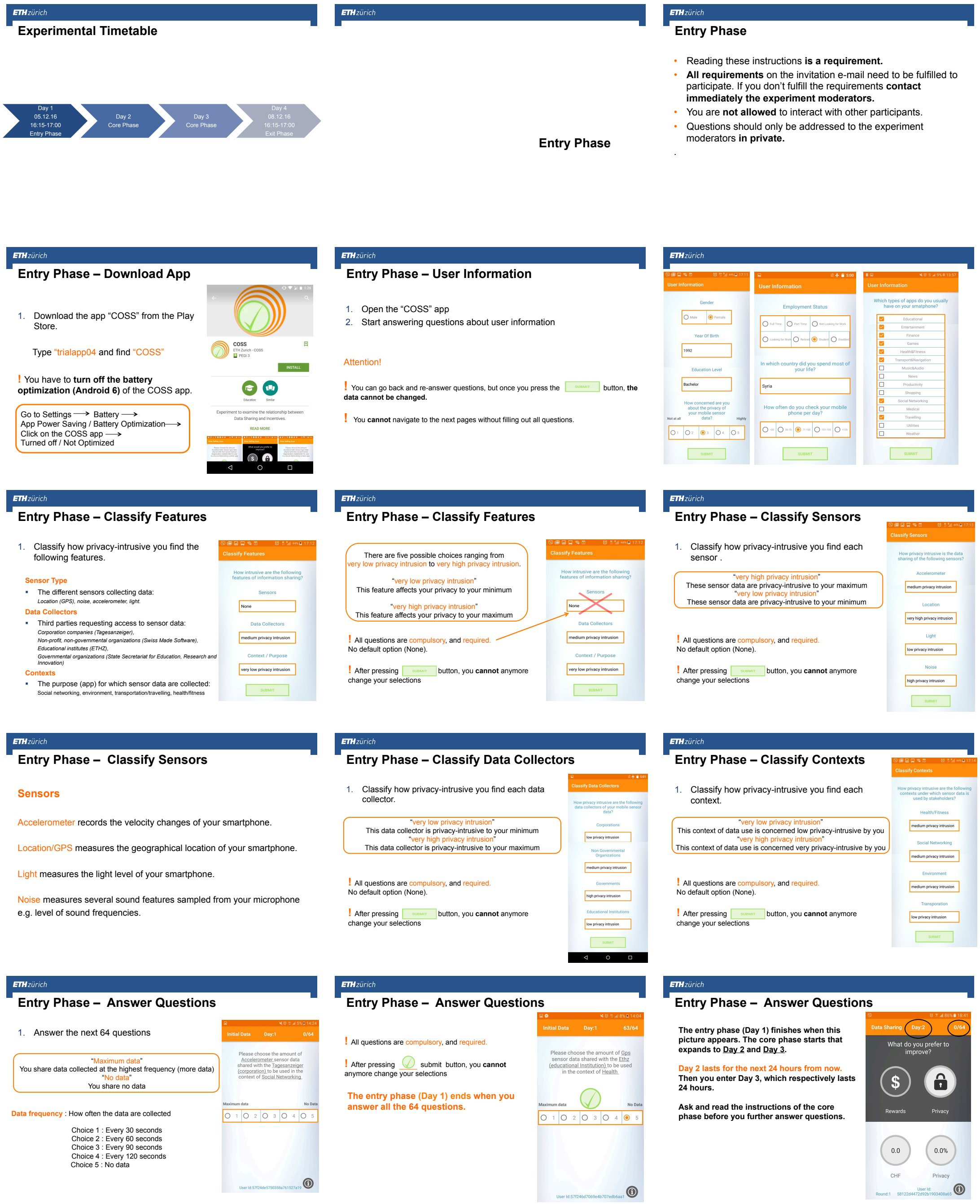

Figure S1: Instructions presented to the participants starting with the entry phase.

participants in the experiment as well as in the data collected in the database. This ID can be viewed in the app by participants. The first screens of the app present the survey questions B.1-B.8

General points:

This study aims at studying the perception people have about privacy and information sharing in the context of mobile sensor data. Nowadays, smartphones are equipped with sensors that can collect real-time information such as our GPS location, the acceleration of motion or even environmental information, for instance temperature and humidity. Smartphones run applications (apps) that are pieces of software with potential access to sensor data. This data can be shared with remote stakeholders such as companies, governments, educational institutions, and others.

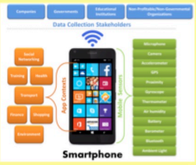

The analysis of mobile sensor data by these stakeholders may put privacy at risk, especially when the sensor data is collected with a fine-grained frequency. Therefore, the amount of mobile sensor data that a user chooses to share with a certain stakeholder for a certain purpose (app context) indicates his/her preferred privacy settings. Participants can assume that any security requirement is met and privacy is entirely governed by their decisions.

Stakeholders can access the sensor data of participants via the web portal fair-data-share.inn.ac. Access to the data complies to the decisions that participants make during the experiment. Stakeholders agree to neither share the data that they can potentially access via the web portal nor infer any individual from the values of sensor data.

**Mandatory components:**	**Annotation:**
a) Goals of the study	Understand human perception on privacy of mobile sensor data and how this perception influences online decision-making about sharing sensor data. Moreover, this study aims at understanding how decision-making is influenced when incentives, e.g. monetary ones, are given to citizens in order to share a higher/lower amount of sensor data at a cost of lower/higher privacy-preservation respectively.
b) Research procedure (methods)	A social experiment requiring a 2-day participation at the ETH Decision Science Lab and 2-day usage of a mobile app.
c) Schedule	The social experiment is outlined in 3 phases:<br>1. Entry phase (45 mins work): Show up at the ETH Decision Science Lab, instructions, sign of information consent, app installation, entry app survey<br>2. Core phase (45 mins work): A two-day app usage.<br>3. Exit phase (30 mins work): Show up at the ETH Decision Science Lab, exit web survey, receipt of rewards.
d) Conditions to be met for participation in the study	Participation in this study requires the following:<br>1. having a general interest and concerns about privacy<br>2. having a smartphone running Android<br>3. having a mobile internet connection<br>4. coming up to the entry phase with a fully charged phone<br>5. committed to participate in all following three phases of the experiment.
e) Advantages and disadvantages for participants / Risks	This research is dedicated to a better understanding of privacy. The findings can be potentially used to improve privacy awareness and preservation of citizens. All risks related to participants' anonymity, information leak, malfunctions and data loss are minimized.
f) Source of funding	This study is supported by the European Community's H2020 Program under the scheme 'INFRAIA-1-2014-2015: Research Infrastructures', grant agreement #654024 'SoBigData: Social Mining & Big Data Ecosystem' (http://www.sobigdata.eu)
g) Compensation/Reimbursement	Participants will be reimbursed with a maximum of 75 CHF as follows:<br>• 20 CHF entry fee for the entry and exit phase<br>• 20 CHF participation fee for the entry and exit phase<br>• 5 CHF app use fee for the core phase<br>• 30 CHF of max rewards based on the received answers
h) Right of withdrawal	As a participant, you have the right to withdraw from the study at any time without needing to specify any reasons or facing negative consequences.
i) Data protection	The anonymity of the participants is guaranteed throughout the experimental process and later on during the data analysis. Participants will be have a unique identifier. The participants' responses and sensor data collected are bound to this unique identifier and there is no other link to any personal information. The data leaving the phone are encrypted and stored in a secure server. The data collected can be processed and analyzed within the Computational Social Science group for research purposes. The stakeholders can potentially access the sensor data that each participant permitted during the experiment and they are not allowed to share any further these data.
j) Insurance coverage	Possible damages to your health, which are directly related to the study and are demonstrably the fault of ETH Zurich, are covered by the general liability insurance of ETH Zurich (insurance policy no. 100.001 of the Swiss Mobiliar insurance company). However, beyond the before mentioned, the health insurance and the accident insurance (e.g. for the way to or back from the study location) is in the responsibility of the participant.
k) Contact person(s)	Dr. Evangelos Pournaras – epournaras@ethz.ch

⇒ ***Please read this form carefully.***
⇒ ***Please ask the investigator or the contact person if you have any questions.***

Study title: Inventivized and privacy-preserving sharing of mobile sensor data – A social experiment

Study location: ETH Zurich, Professorship of Computational Social Science, Clausiusstrasse 50, 8092, Zurich, Switzerland

Principal Investigator's Name and First Name*: Prof. Dr. Dirk Helbing and Dr. Evangelos Pournaras*

Participant's Name and First Name:

Participant:

⇒ I participate in this study on a voluntary basis and can withdraw from the study at any time without giving reasons and without any negative consequences.
⇒ I have been informed orally and in writing about the aims and the procedures of the study, the advantages and disadvantages as well as potential risks.
⇒ I have read the written information for the volunteers. My questions related to the study participation have been answered satisfactorily. I have been given a copy of the information for the volunteers and the consent form.
⇒ I was given sufficient time to make a decision about participating in the study.
⇒ With my signature I certify that I fulfill the requirements for the study participation mentioned in the information for the volunteers.
⇒ I have been informed that possible damages to my health which are directly related to the study and are demonstrably the fault of ETH Zurich, are covered by the general liability insurance of ETH Zurich (insurance policy no. 100.001 of the Swiss Mobiliar insurance company). However, beyond the before mentioned, my health- and/or accident insurance (e.g. for the way to or back from the study location) will apply.
⇒ I agree that the responsible investigators and/or the members of the ethical committee have access to the original data under strict confidentiality.
⇒ I am aware that during the study I have to comply with the requirements and limitations described in the information for the volunteers. In my own health interest the investigators can, without mutual consent, exclude me from the study.
⇒ I agree the involved stakeholders of this study to have access to the mobile sensor data I will share according to the choices I will make during this study.

Location, date . . . . Signature volunteer . . . .

Location, date . . . . Signature investigator . . . .

Figure S2: Information consent for participation in the designed experiment.

of Table S4.

The next screens personalize the sharing of sensor data. Initially, the three criteria of (i) sensor type, (ii) data collector and (iii) context receive their weights according to the perception of each participant on how privacy intrusive they are. The answers of the group Question B.9 in the

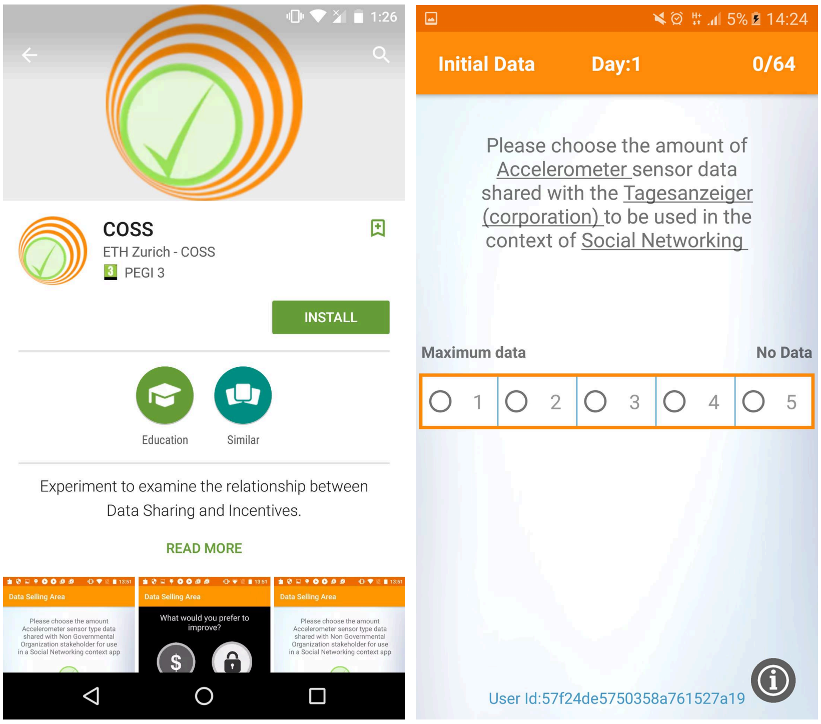

(a) The Android app at Google Play

(b) An instantiation of the factorial question

Figure S3: Screens of the Android app during the entry phase.

range 'very low' to 'very high' are mapped to the weights $w_{i,u}$ of the three criteria as illustrated in Section 1.2. Moving to the next screens, the same personalization process is repeated within each criterion: for the different sensor types (group Question B.10), data collectors (group Question B.11) and contexts (group Question B.12).

Table S5 illustrates the three criteria and its elements during the experiment. For each feature, four possible elements are selected in the factorial experiment to keep a manageable number of $4 * 4 * 4 = 64$ total combinations.

The elements of the data-sharing criteria are chosen after scrutinizing the responses received by the participants of the preparatory phase in Question A.9 for the sensor types (Figure S4a) as well as Question A.13 and A.6 for the contexts (Figure S4b and S4c respectively). Two out of the top-3 highly privacy-intrusive sensors are selected. These are the GPS (privacy intrusion of 0.85) and microphone (privacy intrusion of 0.78). The camera sensor is ranked 2nd with privacy intrusion of 0.83. It is not selected as it requires the collection of more complex data and higher storage space in the smartphones. The accelerometer (ranked 6th with privacy intrusion of 0.47) and light (ranked 7th with privacy intrusion of 0.46) sensors are the other two ones selected that belong into the middle ranking range of privacy intrusion.

Figure S3b illustrates an instantiation example of the factorial question. After answering all questions, participants complete their participation in the entry phase and the smartphone app initializes the core phase. They receive the instructions of the core phase and they depart from DeSciL. Note that the answers to the instantiations of the factorial question during the entry phase

Table S4: Survey questions for the entry phase.

ID	Question	Type	Options
B.1	Gender	multiple choice, one selection	male, female
B.2	Year of birth	multiple choice, one selection	81 [1920,2000]
B.3	Education level	multiple choice, one selection	less than high school, high school, some college, bachelors degree, masters degree, PhD degree
B.4	How concerned are you about the privacy of your mobile sensor data?	ratio scale	5 [not at all,highly]
B.5	Employment status	multiple choice, one selection	full time, part time, not looking for work, looking for work, retired, student, disabled
B.6	In which country did you spend most of your life?	multiple choice, one selection	all countries
B.7	How often do you check your mobile phone a day?	multiple choice, one selection	<35, 36-70, 71-100, 101-135, >135
B.8	Which types of apps do you usually have on your smartphone?	multiple choice, multiple selections	education, entertainment, finance, game, health & fitness, transportation & navigation, music & audio, news, productivity, shopping, social networking, medical, traveling, utilities, weather
B.9	How intrusive are the following features of information sharing?	group of questions	3 questions
B.9.1	Sensors	multiple choice, single selection	very low, low, medium, high, very high
B.9.2	Data collectors	multiple choice, single selection	very low, low, medium, high, very high
B.9.3	Context/Purpose	multiple choice, single selection	very low, low, medium, high, very high
B.10	How privacy intrusive is the data sharing of the following sensors?	group of questions	4 questions
B.10.1	Accelerometer	multiple choice, single selection	very low, low, medium, high, very high
B.10.2	Location	multiple choice, single selection	very low, low, medium, high, very high
B.10.3	Light	multiple choice, single selection	very low, low, medium, high, very high
B.10.4	Noise	multiple choice, single selection	very low, low, medium, high, very high
B.11	How privacy intrusive are the following data collectors of your mobile sensor data?	group of questions	4 questions
B.11.1	Corporations	multiple choice, single selection	very low, low, medium, high, very high
B.11.2	Non-governmental Organizations	multiple choice, single selection	very low, low, medium, high, very high
B.11.3	Governments	multiple choice, single selection	very low, low, medium, high, very high
B.11.4	Educational Institutes	multiple choice, single selection	very low, low, medium, high, very high
B.12	How privacy intrusive are the following contexts under which sensor data is used by stakeholders?	group of questions	4 questions
B.12.1	Health/Fitness	multiple choice, single selection	very low, low, medium, high, very high
B.12.2	Social Networking	multiple choice, single selection	very low, low, medium, high, very high
B.12.3	Environment	multiple choice, single selection	very low, low, medium, high, very high
B.12.4	Transportation	multiple choice, single selection	very low, low, medium, high, very high

are not monetary rewarded. The answers to these questions are the baseline with which the rewarded

Table S5: The selected elements in the criteria for sharing mobile sensor data.

Sensor Type	Data Collector	Context
GPS	Corporation (Tagesanzeiger)	Social networking
Microphone	Non-profit, non-governmental organizations (Swiss Made Software)	Environment
Accelerometer	Educational institutes (ETH Zurich)	Transportation/traveling
Light	Governmental organizations (The State Secretariat for Education, Research and Innovation)	Health/fitness

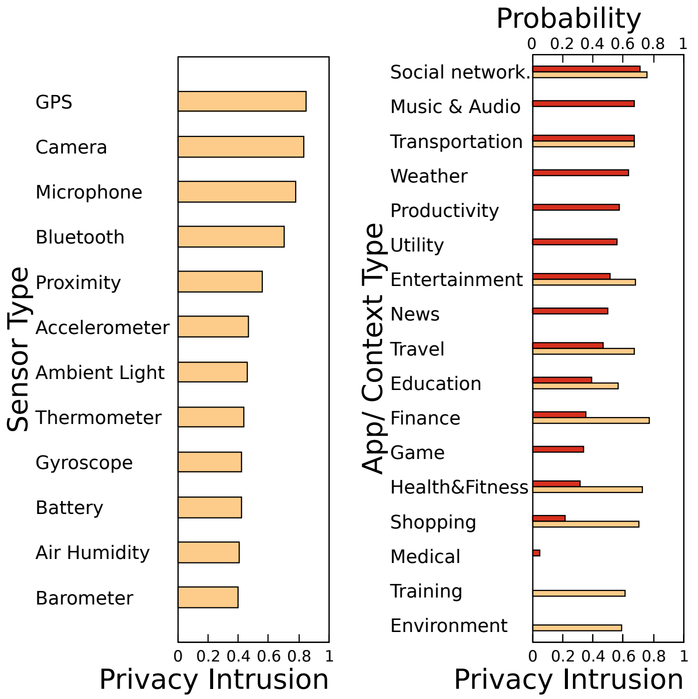

(a) Sensor types (b) Contexts

Figure S4: Data used from Question A.9, A.13 (yellow bars, intrusion) and A.6 (red bars, probability) of the preparatory phase to choose the elements of the data-sharing criteria for the factorial experiment.

sharing of mobile sensor data during the core phase is compared.

3.3 Core phase

The core phase is initialized right after the completion of the entry phase when participants also receive the instructions shown in Figure S5. They also receive at this phase the instructions about the data-access portal, see Figure S6. The core phase lasts for two full days (48 hours, Mondays to Tuesdays and Tuesdays to Wednesdays as shown in Table S2.). It takes place out of DeSciL lab and integrates to the daily life of participants. At the beginning of each day in the core phase, the rewards are zero as no data sharing is performed unless the participants consent to this via their responses to the data-sharing scenarios.

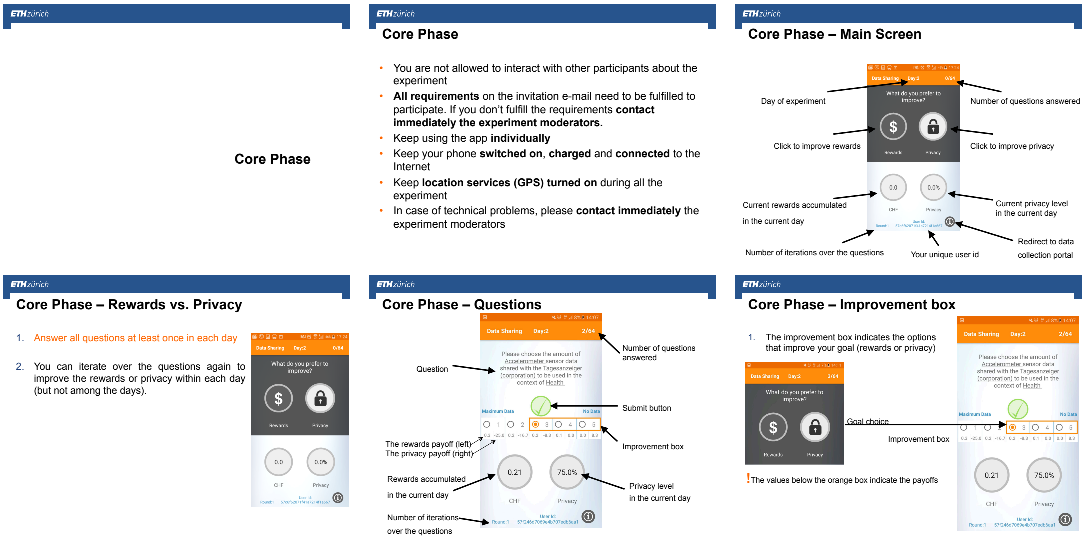

Figure S5: Instructions presented to the participants starting the core phase and after finishing the entry phase.

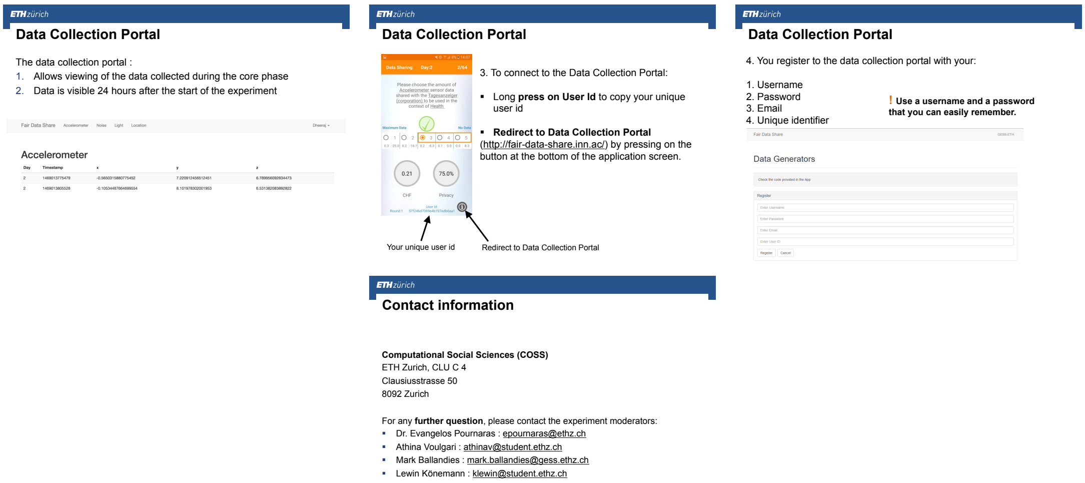

Figure S6: Instructions on the data-access portal presented to the participants starting the core phase and after finishing the entry phase.

3.4 Exit phase

The exit phase is performed on Thursdays, the 4th day of each experimental session (see Table S2), and involves the return of the participants to DeSciL. The staff members of the lab verify the identify of the participants and they are then seated to lab computers to fill in an online survey created in Qualtrics. The questions of the exit survey are outlined in Tables S6 to S9. The matching of the

data collected in this phase with the data of the previous phases is performed with the user ID inserted in Question D.1.

Table S6: General survey questions for the exit phase.

ID	Question	Type	Options
D.1	Please enter your unique ID number	open-ended	-
D.2	Which operating system and which version does your phone have (e.g. Android 6.0.1)?	open-ended	-
D.3	Which mobile phone model have you used for this experiment?	open-ended	-

Table S7: Survey questions for the exit phase–user interface and mobile app functionality.

ID	Question	Type	Options
D.4	How easy was it to use the mobile app of the experiment?	ratio scale	5 [extremely difficult,extremely easy]
D.5	How would you rate the quality of the app?	ratio scale	5 [extremely bad,extremely good]
D.6	How satisfied are you with each of the following features of the mobile app?	group of questions	7 questions
D.6.1	Battery consumption	ratio scale	5 [very low,very high]
D.6.2	Performance speed	ratio scale	5 [very low,very high]
D.6.3	Colors	ratio scale	5 [very low,very high]
D.6.4	Formulation of questions	ratio scale	5 [very low,very high]
D.6.5	Content of questions	ratio scale	5 [very low,very high]
D.6.6	Number of different questions	ratio scale	5 [very low,very high]
D.6.7	Frequency of the notifications	ratio scale	5 [very low,very high]
D.7	Please evaluate the following features of the mobile app:	group of questions	12 questions
D.7.1	How comprehensible was the indicator of the rewards accumulated? (Arrow 1)	ratio scale	5 [very little,very much]
D.7.2	How useful was the indicator of the rewards accumulated to make a choice? (Arrow 1)	ratio scale	5 [very little,very much]
D.7.3	How comprehensible was the indicator of the total privacy level? (Arrow 2)	ratio scale	5 [very little,very much]
D.7.4	How useful was the indicator of the total privacy level to make a choice? (Arrow 2)	ratio scale	5 [very little,very much]
D.7.5	How comprehensible was the indicator of rewards payoff for each data-sharing level? (Arrow 3)	ratio scale	5 [very little,very much]
D.7.6	How useful was the indicator of rewards payoff for each data-sharing level to make a choice? (Arrow 3)	ratio scale	5 [very little,very much]
D.7.7	How comprehensible was the indicator of privacy payoff for each data-sharing level to make a choice? (Arrow 4)	ratio scale	5 [very little,very much]
D.7.8	How useful was the indicator of privacy payoff for each data-sharing level to make a choice? (Arrow 4)	ratio scale	5 [very little,very much]
D.7.9	How comprehensible were the five options of data sharing? (Arrow 5)	ratio scale	5 [very little,very much]
D.7.10	How useful were the five options of data sharing? (Arrow 5)	ratio scale	5 [very little,very much]
D.7.11	How comprehensible was the improvement box? (Arrow 6)	ratio scale	5 [very little,very much]
D.7.12	How useful was the improvement box to make a choice? (Arrow 6)	ratio scale	5 [very little,very much]
D.8	Do you have any other comments regarding the indicators?	open-ended	-

Table S8: Survey questions for the exit phase–privacy and rewards.

ID	Question	Type	Options
D.9	Please evaluate the following questions about privacy:	group of questions	4 questions
D.9.1	Did the experiment make you feel more aware of the privacy of mobile sensor data?	ratio scale	5 [definitely not,definitely yes]
D.9.2	Did the values of privacy represent well your choices of privacy-preservation?	ratio scale	5 [definitely not,definitely yes]
D.9.3	Could you easily adjust your total privacy when it was not satisfactory?	ratio scale	5 [definitely not,definitely yes]
D.9.4	Did your privacy-preservation choices deserved the sacrifice of rewards?	ratio scale	5 [definitely not,definitely yes]
D.10	How satisfied are you with the following?	group of questions	2 questions
D.10.1	The total available amount of rewards (30 CHF)	ratio scale	5 [extremely dissatisfied,extremely satisfied]
D.10.2	The amount of rewards you gained during experiment out of the total available amount of rewards	ratio scale	5 [extremely dissatisfied,extremely satisfied]
D.11	Please evaluate the following statements about rewards:	group of questions	5 questions
D.11.1	Did rewards convince you to share mobile sensor data?	ratio scale	5 [definitely not,definitely yes]
D.11.2	Did rewards convince you to share more mobile sensor data than without rewards?	ratio scale	5 [definitely not,definitely yes]
D.11.3	Did rewards make you more aware about the privacy of mobile sensor data?	ratio scale	5 [definitely not,definitely yes]
D.11.4	Did rewards make you more aware about the value of mobile sensor data?	ratio scale	5 [definitely not,definitely yes]
D.11.5	Did rewards choices deserved the sacrifice of privacy?	ratio scale	5 [definitely not,definitely yes]
D.12	Evaluate the change in rewards payoff (Arrow 3) among the different data-sharing options	ratio scale	5 [very low,very high]
D.13	Evaluate the change of privacy level payoff (Arrow 4) among the different data-sharing options.	ratio scale	5 [very low,very high]

The exit survey begins by acquiring general information about the mobile phone used during the experiment as shown in Table S6. Questions about the user interface and functionality of the mobile app are posed (Table S7). The ease of use and the quality of the app are evaluated in Questions D.4 and D.5 respectively, with the group Question D.6 evaluating the satisfaction level of several features

Table S9: Survey questions for the exit phase–experiment

ID	Question	Type	Options
D.14	Have you participated before in the following:	group of questions	3 questions
D.14.1	An experiment at ETH Decision Science Lab?	multiple choice, one selection	yes, no
D.14.2	A social experiment elsewhere?	multiple choice, one selection	yes, no
D.14.3	An experiment that requires the use of a mobile app?	multiple choice, one selection	yes, no
D.15	How interesting was the experiment?	ratio scale	5 [not interesting at all,extremely interesting]
D.16	Would you participate in a similar experiment again?	ratio scale	5 [definitely not,definitely yes]
D.17	How satisfied are you with the following:	group of questions	6 questions
D.17.1	The written instructions given during the experimental process	ratio scale	5 [extremely dissatisfied,extremely satisfied]
D.17.2	Your participation in the entry phase	ratio scale	5 [extremely dissatisfied,extremely satisfied]
D.17.3	Your participation in the core phase	ratio scale	5 [extremely dissatisfied,extremely satisfied]
D.17.4	Your participation in the exit phase	ratio scale	5 [extremely dissatisfied,extremely satisfied]
D.17.5	The technical support of the staff members moderating the experiment	ratio scale	5 [extremely dissatisfied,extremely satisfied]
D.17.6	Your participation in the overall experiment	ratio scale	5 [extremely dissatisfied,extremely satisfied]
D.18	Has your mobile phone been turned off during the experiment?	multiple choice, one selection	yes, no
D.19	Have you run out of battery during the experiment?	multiple choice, one selection	yes, no
D.20	If yes, please provide some more information (e.g. how long, how many times, at what time of the day)	open-ended	-
D.21	Which of the following reasons prevented you from answering more questions?	multiple choice, multiple selection	I was not interested anymore, I was not enough motivated, I faced technical problems, I ran out of battery, I was busy, I was not satisfied by the experiment, I was concerned about my privacy, other
D.22	Did you think at any time to drop out of the experiment?	multiple choice, one selection	yes, no
D.23	If yes, what was the reason?	open-ended	-
D.24	Did you experience any of the following technical problems?	multiple choice, multiple selection	application crashed, application froze, application was too slow, network connection problems, battery drain, other
D.25	Have you been aware of the Data Collection Portal?	multiple choice, one selection	yes, no
D.26	Have you ever visited the Data Collection Portal?	multiple choice, one selection	yes, no
D.27	How many times did you visit the Data Collection Portal?	multiple choice, one selection	never, less than three times, more than 3 times
D.28	Did you know about this experiment before participating?	multiple choice, one selection	yes, no

such as colors, formulation of questions, number of questions and others. The group Question D.7 evaluates how comprehensible and useful the user interface features are (Figure 9 in the main paper). These questions are used to detect possible biases that may affect data-sharing choices. The questions of Table S8 follow that concern the rewards and privacy. A few factors evaluated are the awareness about privacy (Question D.9.1), ease of privacy adjustments (Question D.9.3), satisfaction level on rewards (Question D.10), data-sharing incentivization by rewards (Question D.11) and other. These questions further explain the data-sharing choices made during the entry and core phase. Table S9 includes the following questions about the experimental process. They evaluate the satisfaction level to several experimental aspects (Question D.17), the participation level and technical problems (Questions D.18-D.24) as well as the user experience of the data-access portal (Questions D.25-D.27).

After the exit survey, participants have an interview with the moderators of the experimental session. The goal of the interview is to scrutinize in a more qualitative way how participants perceive the overall experimental process as well as to discuss some behavioral artifacts observed in the data collected by the Kinvey backend during the previous phases. Moreover, when data are not successfully transferred to Kinvey, the data are manually transferred from the participants' phones to the moderators' computers after participants' consent. At the end of the interview, the

moderators compute and validate the final total compensation of each participant, who receives the compensation by the lab moderators before departing from DeSciL.

3.5 Compensation and monetary incentives

The computed rewards are personalized according to the model of Section 1. The entry phase receives higher compensation as it requires the initial engagement and the execution of more complex tasks with the smartphone compared to the exit phase.

The distribution of the rewards for the app use follows a geometric progression and is implemented by transforming Equation 7 as follows:

$$r_{i,j} = \hat{r}_{i,j} \cdot \sqrt[z-1]{\frac{B_{\mathsf{p}}}{B}}^{s_{i,j}-1}. \tag{9}$$

where $\hat{r}_{i,j}$ is the maximum rewards that can be gained in sharing scenario j computed by Equation 5, $z = 5$ is the number of sharing options, $s_{i,j}$ is the participant's selection, B_{p} is the participation budget and B is the total available budget.

The allocated amounts for the compensation of participants are decided empirically after consultation with the DeSciL staff members. Factors that influence the decisions are the following: the available budget, the target of employing around 100 participants, the complexity of the designed experimental process, Swiss economy and the student profile of the participants in the DeSciL pool. The amounts reflect a trade-off: high enough to inventivize and engage participants with this novel experimental process while not too high to study data-sharing dilemmas between privacy and monetary rewards. The effectiveness of the selected amounts is evaluated using Questions D.9-D.13 of Table S8. These results show that the designed rewards were effective for their purpose. A 57.7% of the participants were too busy to answer more questions, while 33.6% needed further motivation (Question D.21).

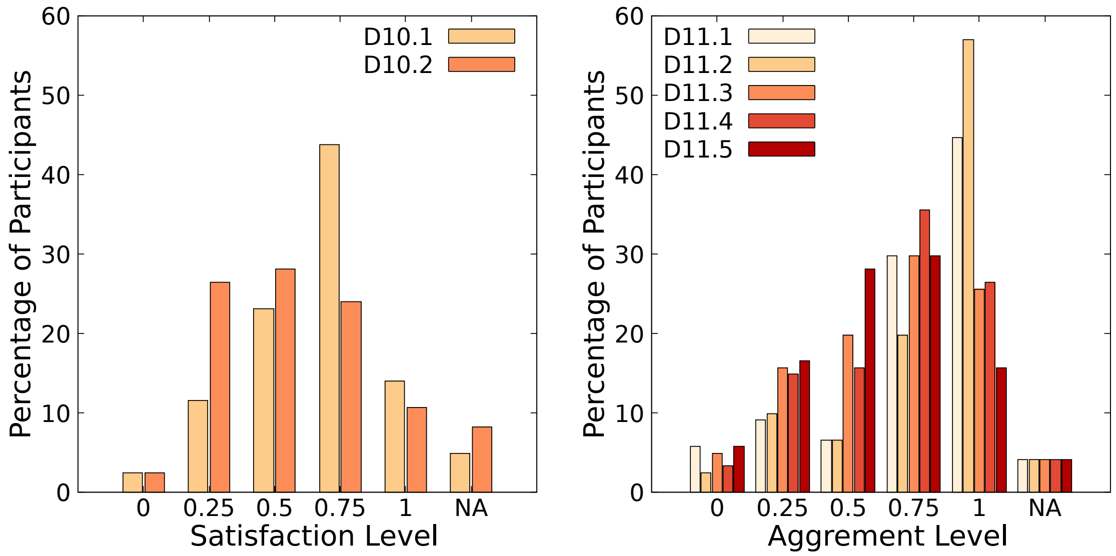

Figure S7: The assessment of the received rewards by the participants of the experiment using group Questions D.10 and D.11.

4 Implementation of the Technical Infrastructure

The data collected by participants' smartphone app are stored and managed locally by an implementation of the nervousnet framework [2] that provides high-level application programming interfaces (APIs) to store, query and analyze data on smartphones. Remotely on the server, the data are stored and managed by Kinvey [3] that provides secure communication by using TLS/SSL encryption between smartphones and the Kinvey backend. The data-access web portal relies on Node.js and a MongoDB database.

The quality of the app (Question D.5) is evaluated 61% positively. The mobile phone remained turned on during the experiment in 82.7% of the participants (Question D.18), while only 13.8% of the participants ran out of battery (Question D.19) and a 25.9% reported battery drain problems (Question D.24).

5 The Privacy and Rewards Gain of Data-sharing Scenarios

Figure S8 illustrates the mean privacy and reward gain of the data-sharing scenarios retrieved as a response of choosing to improve privacy and rewards respectively (see Figure 9 in the main paper).

Table S10 outlines the mean privacy and reward gain of the different data-sharing elements that consist the 64 data-sharing scenarios.

Table S10: Mean privacy and reward gain of the different data-sharing elements involved in the data-sharing scenarios.

Mean Gain	acc	lig	noi	gps	cor	ngo	gov	edu	soc	env	tra	hea
Privacy	4.75	1.92	1.4	1.36	3.13	2.56	2.02	1.71	2.5	2.3	2.29	2.33
Reward	0.16	0.16	0.16	0.19	0.18	0.16	0.17	0.16	0.18	0.16	0.16	0.16

6 Privacy Loss and Rewarded Data-sharing Choices of Groups

Figure S9a and S9b illustrate the probability and cumulative density functions for the intrinsic, 1st and 2nd rewarded data sharing. The two experimental conditions for rewarded data sharing show very similar densities, while intrinsic data sharing comes with a single peak around the privacy level of 0.55.

Figure S9c shows the privacy level over consecutive data-sharing choices under the 2nd rewarded data sharing. Compared to Figure 7b in the main paper showing the 1st rewarded data sharing, the group behaviors are similar. Reward opportunists show a further decline of their privacy level.

7 Goal Signals for Coordinated Data Sharing

Figure S10 illustrates the five goal signals of privacy preservation. They represent a distribution of the required amount of data over the 64 data-sharing scenarios. They are referred within the range of very high to very low privacy preservation. This is because each signal measures the ratio of participants that choose a certain data-sharing level for each data-sharing scenario under intrinsic data sharing. Note that for each data-sharing scenario in Figure S10, the shares of participants sum up to 1.

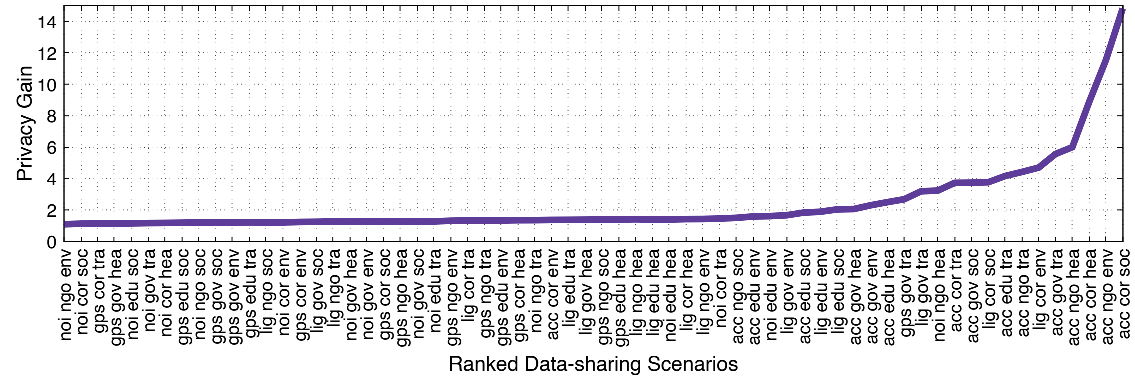

(a) Privacy gain of data-sharing scenarios

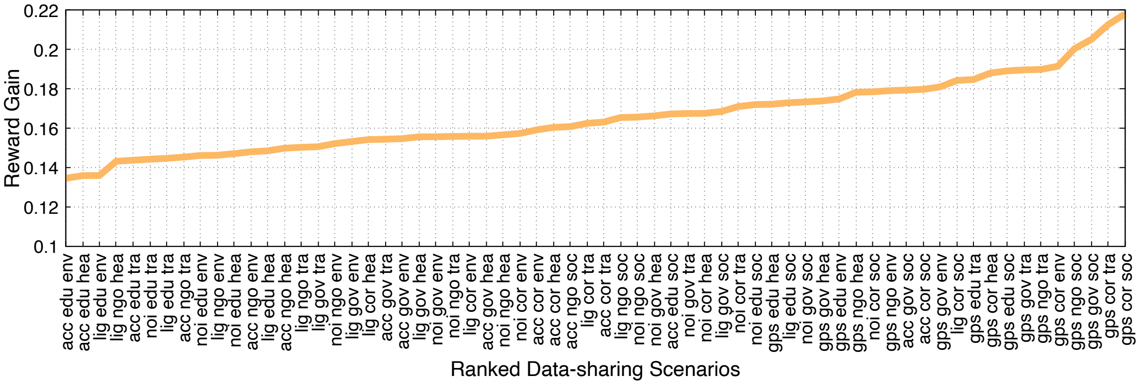

(b) Reward gain of data-sharing scenarios

Figure S8: Mean privacy and reward gain for the 64 data-sharing scenarios under rewarded data sharing. The scenarios are sorted from lowest to highest gain.

8 Data-sharing Mismatch

Figure S11 shows the data-sharing mismatch for the rest of the three goal signals of privacy preservation: low, medium and high. The results here confirm the findings illustrated in Figure 3b of the main paper: mismatch increases for higher privacy-preservation goals as agents mainly have one privacy preserving option (intrinsic) to choose from.

9 Valuations of Collective Privacy Recovery

Four different valuations of privacy are compared in Table S11. All valuations are a function of r_i that is the mean privacy level over all data-sharing scenarios, measured by the gained rewards as outlined in Equation 7:

- **Absolute shared data**: The privacy cost $C_i(r_i)$ equals the gained rewards r_i. This is the default valuation used throughout the main paper. The minimum privacy cost is 0, while the maximum is 17.5 that is the maximum rewards that an individual could gain in the lab

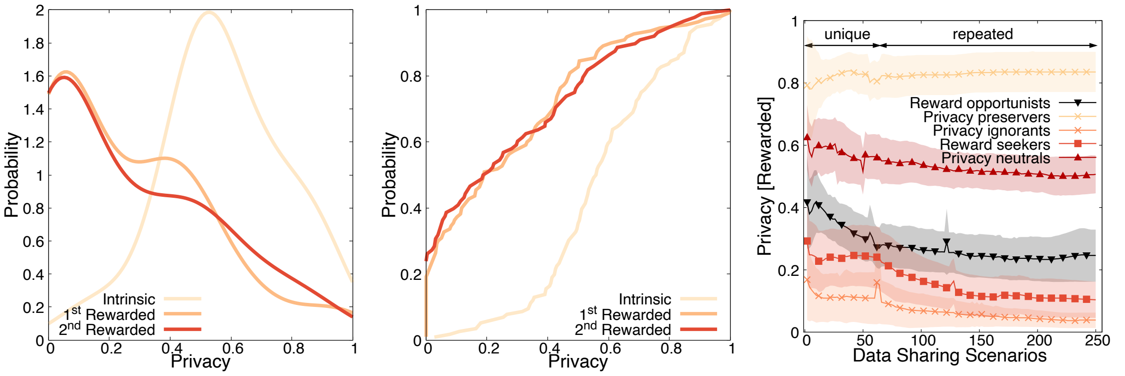

(a) Probability density function of privacy for intrinsic and rewarded data sharing.

(b) Cumulative density function of privacy for intrinsic and rewarded data sharing.

(c) Mean privacy of groups over consecutive rewarded data-sharing choices.

Figure S9: Privacy loss under rewarded data sharing and the behavior of the groups over repeated data-sharing choices under the 2nd rewarded data sharing.

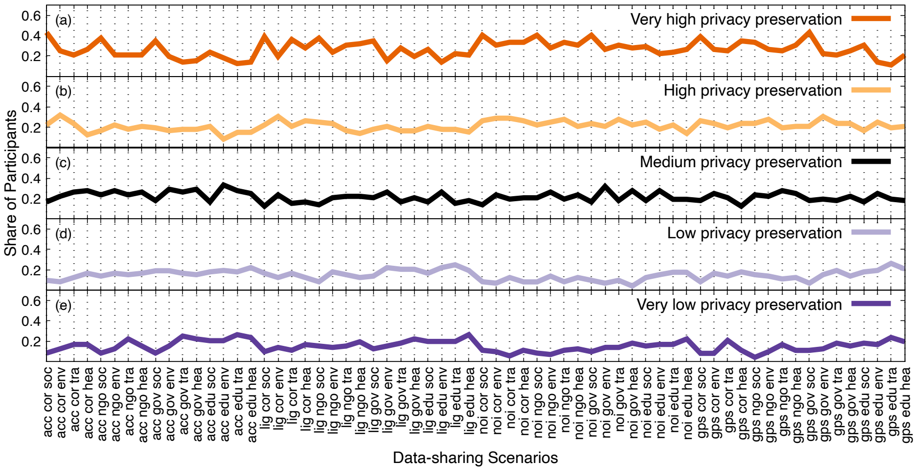

Figure S10: The five goal signals of privacy preservation: from very high to very low. For each signal, the values of a data-sharing scenario measure the share of participants that choose a certain level of privacy preservation.

experiment.

- **Absolute sacrificed rewards**: The privacy cost $C_i(r_i)$ equals the gained rewards r_i minus the fixed data-sharing rewards B_s. This scheme is equivalent to the absolute shared data as B_s is constant. This valuation measures more directly the loss of rewards in exchange of privacy preservation. The minimum privacy cost is -17.5, while the maximum one is 0.

- **Relative shared data**: The privacy cost $C_i(r_i)$ equals the gained rewards r_i minus the privacy level under intrinsic data sharing, measured as well in terms of (hypothetical) gained rewards ($\tilde{r}_i$). The privacy cost of this scheme measures the additional privacy loss under rewarded

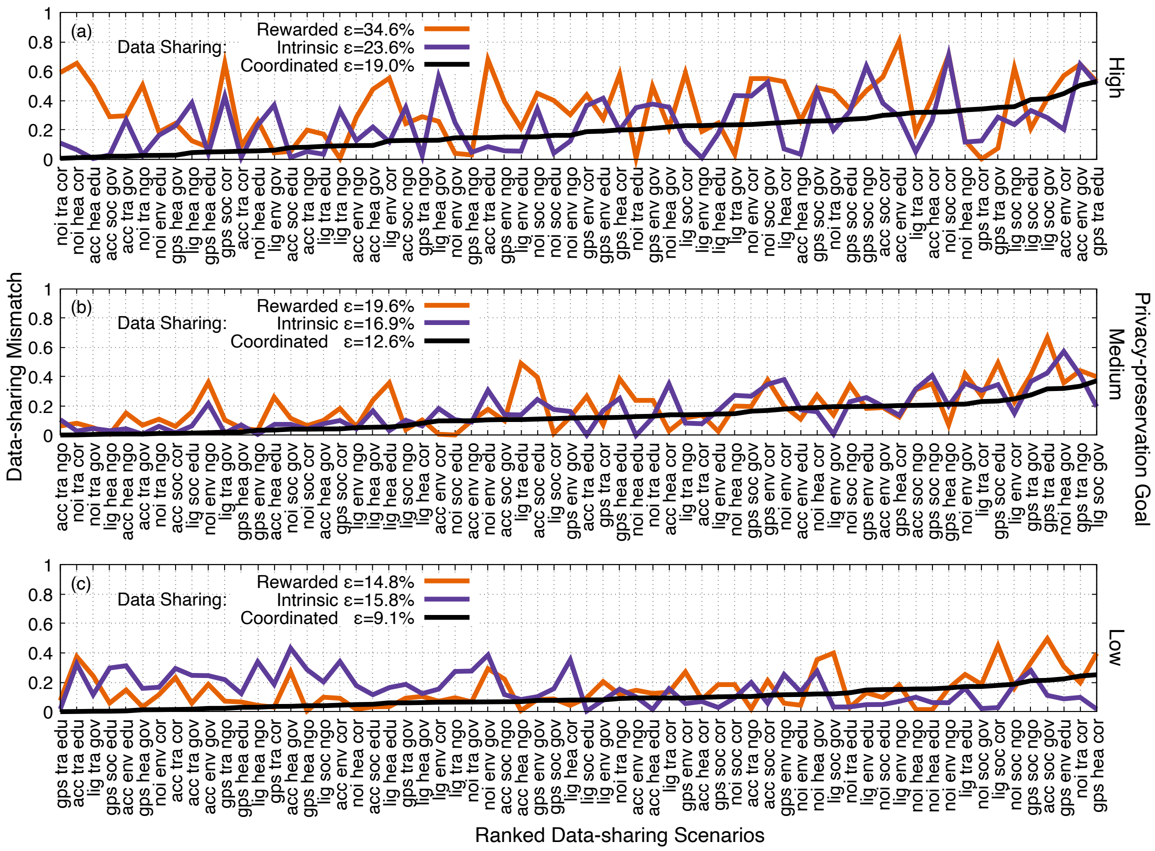

Figure S11: Data-sharing mismatch (root mean square error ε) for the 64 data-sharing scenarios and for the three goal signals of high, medium and low privacy preservation. Values are sorted from lowest to highest mismatch according to the the coordinated data sharing. Coordinated data sharing shows higher efficiency than intrinsic and rewarded data sharing.

Table S11: Four valuation schemes and their range of values.

Valuation	Relation	min/max $\tilde{r}_i$	Without Rewards ($r_i \equiv \tilde{r}_i$)		With Rewards	
			min $C_i(0)$	max $C_i(17.5)$	min $C_i(0)$	max $C_i(17.5)$
Absolute shared data	$C_i(r_i) = r_i$		0	17.5	0	17.5
Absolute sacrificed rewards	$C_i(r_i) = r_i - B_s$		-17.5	0	-17.5	0
Relative shared data	$C_i(r_i) = r_i - \tilde{r}_i$	$\tilde{r}_i = 0$	0	0	0	17.5
		$\tilde{r}_i = 17.5$	0	0	-17.5	0
Relative sacrificed rewards	$C_i(r_i) = r_i - (B_s - \tilde{r}_i)$	$\tilde{r}_i = 0$	-17.5	-17.5	-17.5	0
		$\tilde{r}_i = 17.5$	-17.5	17.5	0	17.5

data sharing over the intrinsic one, assuming that the intrinsic data sharing comes with no privacy cost. Depending on the level of intrinsic data sharing, the minimum privacy cost is -17.5, while the maximum is 17.5 (the behavior of reward opposer and reward opportunist respectively as shown in Table 1 of the main paper).

- **Relative sacrificed rewards**: The privacy cost $C_i(r_i)$ equals the gained rewards r_i minus the privacy preservation under intrinsic data sharing measured by $B_\mathsf{s} - \tilde{r}_i$. This scheme is equivalent to the one of absolute sacrificed rewards with the addition of the privacy cost $\tilde{r}_i$ under intrinsic data sharing. Depending on the level of intrinsic data sharing, the minimum privacy cost is -17.5, while the maximum one is 17.5.

The collective privacy recovery under intrinsic, rewarded and coordinated data sharing is assessed using the four different valuations schemes under the very high and very low privacy preservation goal. Figure S12 shows the privacy cost per individual for each of these cases. All lines are sorted from lowest to highest privacy cost. Each plot in Figure S12 comes with the mean relative privacy gain and loss of the coordinated data sharing compared to rewarded and intrinsic data sharing respectively. The privacy cost of intrinsic data sharing corresponds to the data-sharing plan with the minimum privacy cost and it is calculated using EPOS with $\alpha = 0, \beta = 1$. In contrast, the privacy cost of rewarded data sharing corresponds to the data-sharing plan with the maximum privacy cost and it is calculated using EPOS with $\alpha = 0, \beta = 1$ and data-sharing plans with reversed sign.

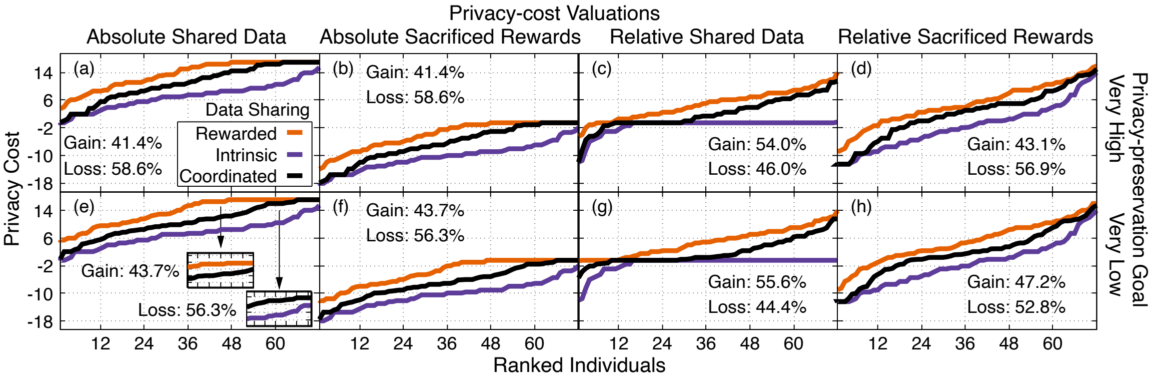

Figure S12: The four privacy valuations illustrated in Table S11. The privacy cost is measured for the intrinsic, rewarded and coordinated data sharing under the very high and very low privacy preservation goal. The highest privacy gain is observed for the relative shared data and the relative sacrificed rewards.

The highest privacy gains are observed under the valuation scheme of relative shared data: 54% and 55.6% for the very high and very low privacy preservation goal. This means that coordinated data sharing shows a further privacy recovery when evaluating the data-sharing choices based on the additional privacy cost that individuals pay over the intrinsic data sharing. The relative sacrificed rewards follow with 43.1% and 47.2% respectively. The default valuation scheme of absolute shared data has the lowest privacy gain of 41.4% and 43.7% respectively, which equals the absolute sacrificed rewards as B_s is constant (lines shifted to negative values). The mean privacy gain for the very low privacy preservation goal is 2.7% higher than the very high one. Similarly with the observation in Figure 3a of the main paper, two rewarded options of individuals with low privacy on average provide higher flexibility than a single one with high privacy preservation.

With their higher privacy gains, the alternative valuation schemes find applicability in the further adoption of the data-sharing plans recommended to users. They can also be used to provide augmented explanations of what these recommended plans mean for the data collective, while raising awareness of the different privacy manifestations and collective privacy gains.

10 Privacy Reinforcement

Figure S13 illustrates the privacy reinforcement of the different data-sharing elements. The key finding is that the perceived privacy sensitivity of the data-sharing elements is likely to reinforce privacy under intrinsic and coordinated data sharing rather than the rewarded ones.

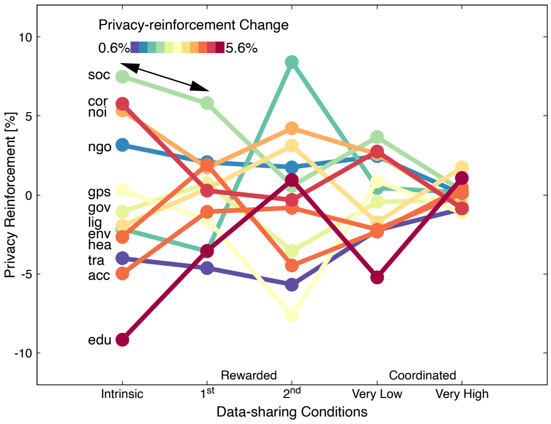

Figure S13: Privacy reinforcement of the different sensors, collectors and contexts under intrinsic, rewarded and coordinated data sharing. The 12 colored lines are ranked according to the reinforcement change (intrinsic - 1st rewarded data sharing).

The mean absolute privacy reinforcement under intrinsic data sharing is higher than 1st rewarded and the two coordinated data-sharing conditions: 4%>2.27%> 2.24%>0.65% respectively. Under intrinsic data sharing, social networking, corporation, noise sensor and NGO reinforce privacy gain, while education, accelerometer and transportation a privacy loss. Privacy reinforcement under intrinsic data sharing is correlated with the attitudinal privacy sensitivity ($R = 0.63, t(10) = 2.57, p = 0.028$). This means that privacy risk awareness is likely to reinforce privacy protection. There is a correlation in the privacy reinforcement under intrinsic and the 1st rewarded data sharing ($R = 0.73, t(10) = 3.4, p = 0.0067$). In the 2nd rewarded data sharing, GPS shifts to a 3.5% reinforcement of privacy loss, while environment shifts to a 8.4% reinforcement of privacy gain. Coordinated data sharing with the very low privacy-preservation goal is positively correlated to attitudinal ($R = 0.65, t(10) = 3.68, p = 0.023$), intrinsic ($R = 0.96, t(10) = 11.58, p = 4.07 \times 10^{-7}$) and the 1st rewarded ($R = 0.62, t(10) = 2.48, p = 0.032$) data sharing. With the very high privacy-preservation goal, the correlation to attitudinal data sharing is negative: $R = -0.61, t(10) = -2.43, p = 0.035$.

11 Conjoint Analysis

The assumptions of conjoint analysis are discussed and assessed in the context of the conducted experiment [4]. No direct carryover effects are involved under instinct data sharing as participants are exposed to each data-sharing scenario once. Under rewarded data sharing, the privacy-rewards balance introduces a carryover effect that is subject of study in this paper. Because rewards are personalized (i.e. each data-sharing scenario is retrieved to satisfy the intended action of improving rewards or privacy) and because responses to repeated data-sharing scenarios are made on-demand, carryover effects mainly originate from tuning the privacy-rewards balance. No influential order effects are anticipated within the designed rating-based conjoint experiment.

In regards to the order of the data-sharing elements, each data-sharing scenario is presented in natural language as determined by the Factorial Question in Section 4.1 of the main paper. Decision-making quality is not expected to decrease for $k = 3 < 10$ data-sharing criteria as shown in earlier experimental tests in literature [5, 4].

As this is not a choice-based conjoint experiment, the order of the data-sharing levels (Figure 9b of the main paper) simply adheres to design principles of likert scales and graphical user interfaces.

As the experiment relies on a full-factorial design without rendering any data-sharing scenario as infeasible, order effects among the scenarios are unlikely. It is though personalization under rewarded data sharing that can yield, in theory, atypical data-sharing choices, i.e. one that can increase the accumulated rewards when a participant chooses to improve privacy, and vice versa. Excluding these or reducing the likelihood of their occurrence is expected to improve external validity [4], i.e. participants do not lose interest or react contrary to their privacy-reward goal improvement.

The performed conjoint analysis relies on the following multiple linear regression model:

$$\lambda_{0,0} + \lambda_{1,1} \cdot \mathcal{D}_{1,1} + ... + \lambda_{1,l_1-1} \cdot \mathcal{D}_{1,l_1-1} + ... + \lambda_{k,l_k-1} \cdot \mathcal{D}_{k,l_k-1} + \epsilon \quad (10)$$

where $\lambda_{u,o}$ for each of the criteria $u \in \{1, ..., k\}$ and elements $o \in \{1, ..., l_k - 1\}$ are the estimated coefficients of the regression model, with $\lambda_{0,0}$ representing the intercept and ϵ the regression error. The $\mathcal{D}_{u,o}$ for each of the criteria $u \in \{1, ..., k\}$ and elements $o \in \{1, ..., l_k - 1\}$ are the independent dummy variables that represent the absence or presence of a data-sharing element within a data-sharing scenario. Note that one data-sharing element for each criterion is removed from the model (accelerometer, corporation, social networking) to resolve the linear dependency with which the effect of the confounded variables cannot be separated by the regression.

Using the estimated coefficients, the partworth utilities can be estimated for each data-sharing criterion u as follows:

$$\mathcal{P}_u = \frac{\max_{o=1}^{l_u} \lambda_{u,o} - \min_{o=1}^{l_u} \lambda_{u,o}}{\sum_{u=1}^{k}(\max_{o=1}^{l_u} \lambda_{u,o} - \min_{o=1}^{l_u} \lambda_{u,o})}. \quad (11)$$

The partworth utilities measure the relative importance of the criteria within a regression model: which of the data type, collector or context is the most important when individuals make data-sharing decisions. Similarly, the relative importance of each data-sharing element for each criterion is calculated as follows:

$$\mathcal{P}_{u,o} = \frac{\lambda_{u,o} - \frac{1}{l_u} \cdot \sum_{o=1}^{l_u} \lambda_{u,o}}{\sum_{u=1}^{k}(\max_{o=1}^{l_u} \lambda_{u,o} - \min_{o=1}^{l_u} \lambda_{u,o})}. \quad (12)$$

The relative importance calculation can be adjusted for each data-sharing element among all criteria as follows:

$$\hat{\mathcal{P}}_{u,o} = \frac{\lambda_{u,o} - \frac{1}{k \cdot l_u} \cdot \sum_{u=1}^{k} \sum_{o=1}^{l_u} \lambda_{u,o}}{\max_{u=1}^{k} \max_{o=1}^{l_u} \lambda_{u,o} - \min_{u=1}^{k} \min_{o=1}^{l_u} \lambda_{u,o}}. \quad (13)$$

The model of Equation 10 is evaluated at the population level for different dependent variables of privacy P_j and rewards R_j with values over the 64 data-sharing scenarios. These variables are selected among the different experimental conditions and they determine the compared conjoint analysis models. The regression coefficients are illustrated in Table S12 and Figure S14. The rest of the conjoint analysis and metrics are shown in Table S13.

Eight models with privacy as the dependent variable as assessed: intrinsic, 1st, 2nd rewarded, intrinsic-1st rewarded, intrinsic-2nd rewarded, 1st rewarded-2nd rewarded, coordinated for very low

Table S12: The coefficients $\lambda_{u,o}$ of nine multiple regression models, each with a different dependent variable of privacy or rewards. The four statistically more powerful models (R^2>0.8) are illustrated in the main paper. These values are used to analyze the relative importance of data-sharing criteria and elements in Table S13.

Models	acc	lig	noi	gps	cor	ngo	gov	edu	soc	env	tra	hea	Intercept
Privacy [Intrinsic]	0	0.023972603	0.087756849	0.043450342	0	-0.023116438	-0.058861301	-0.125856164	0	-0.084974315	-0.099957192	-0.088827055	0.654430651
Privacy [1st Rewarded]	0	0.006753024	0.013699034	-0.001945327	0	0.008754161	0.002140409	-0.018011013	0	-0.045397867	-0.050165302	-0.019421827	0.343937021
Privacy [2nd Rewarded]	0	0.018454293	0.024093745	-0.031555723	0	0.009563823	-0.015594024	0.006242213	0	0.039665329	-0.02786132	-0.022817284	0.310195822
Rewards [1st & 2nd Rewarded]	0	-0.0000117	0.005297903	0.033532185	0	-0.013561209	-0.0074504	-0.018137996	0	-0.022210396	-0.015665036	-0.020453195	0.180147342
Privacy [Intrinsic − 1st Rewarded]	0	0.017219578	0.074057816	0.045395669	0	-0.031870599	-0.061001711	-0.107845152	0	-0.039576448	-0.04979189	-0.069405228	0.31049363
Privacy [Intrinsic − 2nd Rewarded]	0	0.00551831	0.063663104	0.075006065	0	-0.032680261	-0.043267278	-0.132098377	0	-0.124639644	-0.072095871	-0.066009771	0.344234829
Privacy [1st − 2nd Rewarded]	0	-0.011701269	-0.010394712	0.029610396	0	-0.000809662	0.017734433	-0.024253226	0	-0.085063196	-0.022303981	0.003395458	0.033741198
Privacy [Coordinated, very low]	0	0.003576389	0.037118056	0.023663194	0	-0.002256944	-0.024444444	-0.061510417	0	-0.025625	-0.045746528	-0.047048611	0.555911458
Privacy [Coordinated, very high]	0	0.012326389	-0.006076389	-0.009027778	0	0.007118056	0.005034722	0.014930556	0	-0.000868056	-0.008680556	0.001215278	0.489479167

Table S13: Conjoint analysis based on nine multiple linear regression models, each with a different dependent variable of privacy or rewards. The four statistically more powerful models (R^2>0.8) are illustrated in the main paper. The table also shows all calculated partworth utilities (relative importance [%]) and their significance.

Models	Regression statistics		Conjoint statistics	Sensor	Collector	Context	acc	lig	noi	gps	cor	ngo	gov	edu	soc	env	tra	hea
	Multiple R:	0.93	$\mathcal{P}_u$	27.99	40.14	31.88												
Privacy	R^2:	0.86	$\mathcal{P}_{u,o}$				-12.37	-4.73	15.61	1.48	16.57	9.20	-2.20	-23.57	21.83	-5.27	-10.05	-6.50
[Intrinsic]	Adjusted R^2:	0.84	$\hat{\mathcal{P}}_{u,o}$				8.67	16.32	36.66	22.53	8.67	1.30	-10.10	-31.46	8.67	-18.42	-23.20	-19.65
	ANOVA p-value:	5.43×10^{-20}	p-value:				0	0.031	6.67×10^{-11}	1.86×10^{-4}	0	0.037	1.34×10^{-6}	2.55×10^{-16}	0	1.73×10^{-10}	1.07×10^{-12}	4.62×10^{-11}
	Multiple R:	0.78	$\mathcal{P}_u$	16.90	28.91	54.19												
Privacy	R^2:	0.61	$\mathcal{P}_{u,o}$				-5.00	2.30	9.80	-7.10	1.92	11.38	4.23	-17.53	31.05	-17.99	-23.14	10.07
[1st Rewarded]	Adjusted R^2:	0.55	$\hat{\mathcal{P}}_{u,o}$				9.33	16.62	24.12	7.22	9.33	18.78	11.64	-10.13	9.33	-39.71	-44.86	-11.65
	ANOVA p-value:	1.47×10^{-8}	p-value:				0	0.348	0.060	0.786	0	0.225	0.765	0.015	0	4.40×10^{-8}	3.63×10^{-9}	0.009
	Multiple R:	0.85	$\mathcal{P}_u$	37.52	16.96	45.52												
Privacy	R^2:	0.73	$\mathcal{P}_{u,o}$				-1.85	10.59	14.39	-23.13	-0.04	6.41	-10.55	4.17	1.86	28.60	-16.93	-13.53
[2nd Rewarded]	Adjusted R^2:	0.68	$\hat{\mathcal{P}}_{u,o}$				-0.01	12.43	16.23	-21.28	-0.01	6.44	-10.52	4.20	-0.01	26.73	-18.79	-15.39
	ANOVA p-value:	2.77×10^{-12}	p-value:				0	0.033	0.006	4.59×10^{-4}	0	0.263	0.071	0.464	0	1.90×10^{-5}	0.002	0.009
	Multiple R:	0.97	$\mathcal{P}_u$	45.40	24.55	30.06												
Rewards	R^2:	0.93	$\mathcal{P}_{u,o}$				-13.13	-13.15	-5.96	32.25	13.25	-5.11	3.16	-11.30	19.73	-10.32	-1.47	-7.95
[1st & 2nd Rewarded]	Adjusted R^2:	0.92	$\hat{\mathcal{P}}_{u,o}$				6.62	6.60	13.79	52.00	6.62	-11.74	-3.47	-17.93	6.62	-23.44	-14.58	-21.06
	ANOVA p-value:	3.47×10^{-28}	p-value:				0	0.995	6.19×10^{-3}	1.62×10^{-24}	0	1.37×10^{-9}	1.90×10^{-4}	1.63×10^{-13}	0	8.77×10^{-17}	2.05×10^{-11}	2.10×10^{-15}
	Multiple R:	0.85	$\mathcal{P}_u$	29.47	42.91	27.62												
Privacy	R^2:	0.72	$\mathcal{P}_{u,o}$				-13.60	-6.74	15.87	4.47	19.97	7.29	-4.31	-22.95	15.79	0.05	-4.02	-11.82
[Intrinsic − 1st Rewarded]	Adjusted R^2:	0.68	$\hat{\mathcal{P}}_{u,o}$				7.39	14.24	36.86	25.45	7.39	-5.29	-16.89	-35.52	7.39	-8.36	-12.42	-20.23
	ANOVA p-value:	3.65×10^{-12}	p-value:				0	0.194	5.92×10^{-7}	0.001	0	0.018	2.09×10^{-5}	3.99×10^{-11}	0	0.004	3.62×10^{-4}	2.15×10^{-6}
	Multiple R:	0.91	$\mathcal{P}_u$	22.61	39.82	37.57												
Privacy	R^2:	0.82	$\mathcal{P}_{u,o}$				-10.87	-9.20	8.32	11.74	15.68	5.83	2.64	-24.14	19.80	-17.77	-1.93	-0.10
[Intrinsic − 2nd Rewarded]	Adjusted R^2:	0.79	$\hat{\mathcal{P}}_{u,o}$				8.20	9.87	27.39	30.81	8.20	-1.65	-4.84	-31.62	8.20	-29.37	-13.53	-11.69
	ANOVA p-value:	4.95×10^{-17}	p-value:				0	0.681	1.47×10^{-5}	7.10×10^{-7}	0	0.018	0.002	1.04×10^{-13}	0	7.65×10^{-13}	1.57×10^{-6}	7.97×10^{-6}
	Multiple R:	0.83	$\mathcal{P}_u$	24.05	24.45	51.50												
Privacy	R^2:	0.69	$\mathcal{P}_{u,o}$				-1.09	-7.91	-7.15	16.15	1.07	0.60	11.39	-13.05	15.13	-34.39	2.15	17.11
[1st − 2nd Rewarded]	Adjusted R^2:	0.63	$\hat{\mathcal{P}}_{u,o}$				5.04	-1.78	-1.02	22.28	5.04	4.56	15.36	-9.09	5.04	-44.49	-7.95	7.01
	ANOVA p-value:	8.70×10^{-11}	p-value:				0	0.289	0.346	0.009	0	0.941	0.110	0.031	0	2.21×10^{-10}	0.046	0.757
	Multiple R:	0.92	$\mathcal{P}_u$	25.48	42.22	32.30												
Privacy	R^2:	0.84	$\mathcal{P}_{u,o}$				-11.04	-8.59	14.44	5.20	15.14	13.59	-1.64	-27.09	20.32	2.73	-11.08	-11.97
[Coordinated, very low]	Adjusted R^2:	0.81	$\hat{\mathcal{P}}_{u,o}$				8.14	10.59	33.62	24.38	8.14	6.59	-8.64	-34.09	8.14	-9.45	-23.26	-24.16
	ANOVA p-value:	2.52×10^{-18}	p-value:				0	0.544	4.77×10^{-8}	1.68×10^{-4}	0	0.701	1.08×10^{-4}	1.13×10^{-14}	0	5.51×10^{-5}	1.94×10^{-10}	8.48×10^{-11}
	Multiple R:	0.53	$\mathcal{P}_u$	46.24	32.33	21.43												
Privacy	R^2:	0.28	$\mathcal{P}_{u,o}$				1.50	28.20	-11.65	-18.05	-14.66	0.75	-3.76	17.67	4.51	2.63	-14.29	7.14
[Coordinated, very high]	Adjusted R^2:	0.16	$\hat{\mathcal{P}}_{u,o}$				-2.88	23.81	-16.04	-22.43	-2.88	12.53	8.02	29.45	-2.88	-4.76	-21.68	-0.25
	ANOVA p-value:	2.51×10^{-2}	p-value:				0	0.062	0.352	0.169	0	0.276	0.440	0.025	0	0.894	0.185	0.852

and very high privacy preservation. One model with rewards as the dependent variable is assessed: 1st and 2nd rewarded of those individuals who intent and do improve rewards as in Figure 9 in the main paper. In addition, the following four models with the mismatch as dependent variable are assessed: intrinsic, rewarded, coordinated from very low to very high privacy preservation. As they perform statistically poorly, they are not shown in Table S13.

Figure S15 illustrates the relative importance ($\mathcal{P}_u$, $\hat{\mathcal{P}}_{u,o}$) of the data-sharing criteria and elements among all criteria, in contrast to Figure 6 in the main paper that shows the relative importance ($\mathcal{P}_u$, $\mathcal{P}_{u,o}$) of the elements within each criterion.

The relative importance ($\mathcal{P}_u$) of the data-sharing criteria is the same as shown in Figure 6 of the

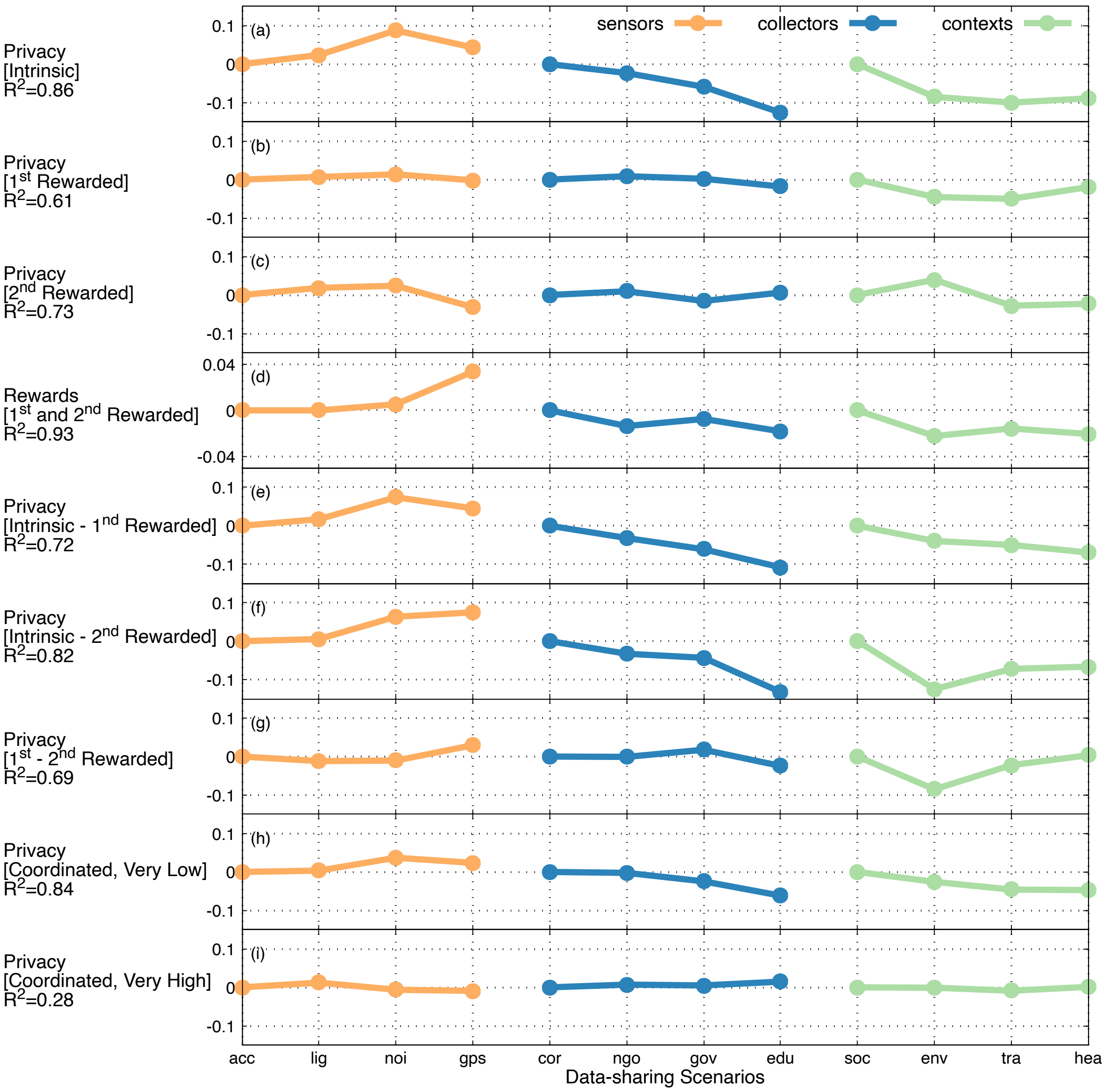

Figure S14: Coefficients of multiple linear regression used in conjoint analysis. Nine models with different dependent variables for privacy and rewards are compared. Four of these models with $R^2 > 0.8$ are shown in the main paper, Figure 6a.

main paper. For all models, sensor data such as GPS (46.82%), noise (41.4%) and light (16.04%) show the highest mean positive relative importance among all elements of the three criteria, while education (-42.74%) from collectors and environment (-29.78%), health (-27.8%) and transportation (-27.35%) from context show the lowest one. In contrast to the regression models, the perceived privacy sensitivity of GPS (44.37%), corporation (27.8%) and social networking (51.44%) are the highest positive ones, while the accelerometer (-44.28%), light (-48.56%) and education (-25.51%)

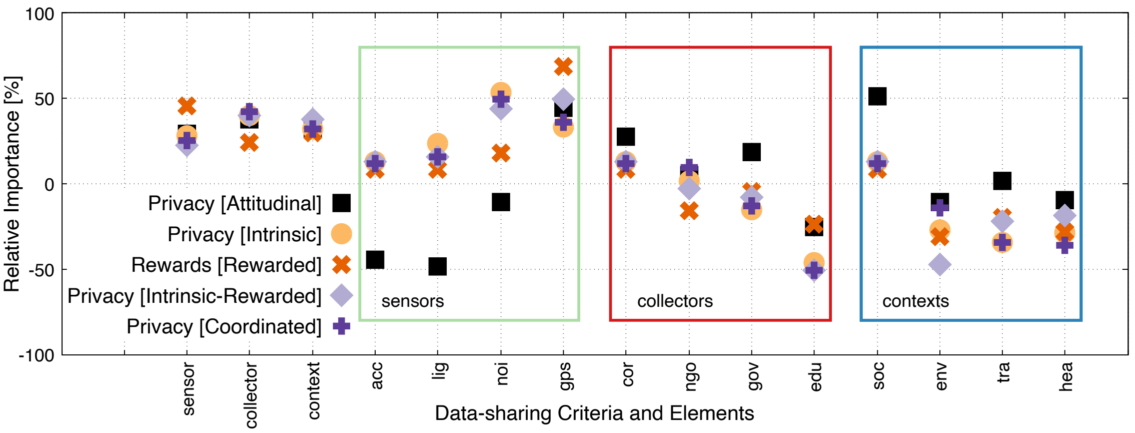

Figure S15: The relative importance (partworth utilities) of the data-sharing criteria and elements (relative among all criteria) derived from the different regression models of conjoint analysis and the perceived privacy sensitivity.

show the highest negative ones.

12 Validation of Groups

Table S14 illustrates the results of the bootstrap evaluation method for the 5 different group behaviors extracted from the experimental data.

Table S14: Results of the bootstrap evaluation method [6] (`clusterboot` of R) for the stability of the clusters. Each entry of results is represented as 'bootmean (bootbrd)', where bootmean is the clusterwise mean Jaccard similaritiy and bootbrd is the clusterwise number of times a cluster is disolved.

Clustering algorithms	k-means	hierachical	pamkCBI
Privacy ignorants	0.79 (8)	0.67 (41)	0.58 (48)
Privacy neutrals	0.93 (0)	0.88 (1)	0.7 (31)
Privacy preservers	0.89 (7)	0.76 (16)	0.7 (31)
Rewards seekers	0.83 (1)	0.75 (17)	0.61 (37)
Rewards opportunists	0.84 (6)	0.76 (14)	0.56 (51)

Furthermore, the split of the participants over the data-sharing groups is compared to privacy categories identified in the general population from studies such as the ones of Westin [7, 8]. This comparison can only be indicative though: a random sample from a US population back in 1990 is compared to a non-random sample from a Swiss population in 2016. Moreover, the survey questions are not identical to the formulated data-sharing prompts. Nevertheless, this comparison has a value out of the the fact that there are groups that capture the intended privacy of a broader population vs. groups that capture the actual data-sharing decisions of typical smartphone users.

Westin's studies classify individuals in three behavioral categories based on survey responses: *privacy fundamentalists, pragmatists and unconcerned.* They cover the whole spectrum of data-

sharing levels depicted in the exemplary of Table 1 in the main paper. Based on this, we match the data-sharing groups to Westin's categories under intrinsic data sharing, i.e. the data-sharing behavior of individuals is not considered under rewarded data sharing. The matching is illustrated in Table S15. The observed groups sizes show a remarkable match to Westin's privacy categories.

Table S15: Matching the Westin's classification [7, 8] to the data-sharing groups without rewards (Table 1 in the main paper).

Westin's population categories [7, 8]		Data-sharing Groups ($n = 84$).	
Privacy fundamentalists	25%	Privacy preservers<br>Reward opportunists	26.2%
Privacy pragmatists	57%	Privacy neutrals<br>Reward seekers	57.14%
Privacy unconcerned	18%	Privacy ignorants	16.7%

13 Analysis of Variance for Data-sharing Criteria and Groups

The Analysis of Variance (ANOVA) is made with IBM SPSS 24.0. Figure S16 summarizes the p values obtained for each data-sharing criterion and its elements. Using the Levene's test, the homogeneity of variances is confirmed ($p > 0.05$) for the majority of the data-sharing criteria and their elements: sensor ($p = 0.169$), data collector ($p = 0.328$), context ($p = 0.956$), GPS ($p = 0.156$), light ($p = 0.896$), noise ($p = 0.432$), corporation ($p = 0.607$), educational institute ($p = 0.35$), government ($p = 0.074$), NGO ($p = 0.993$), health ($p = 0.314$), social networking ($p = 0.486$). It is not confirmed for: accelerometer ($p = 0.04$), transportation ($p = 0.039$) and environment ($p = 0.005$). The whole report analysis is illustrated in Table S16.

The report analysis of the post hoc Tukey's range test ($\alpha = 0.05$) is illustrated in Table S17, S18 and S19.

References

[1] Qualtrics. URL `https://www.qualtrics.com`.

[2] Pournaras, E., Moise, I. & Helbing, D. Privacy-preserving ubiquitous social mining via modular and compositional virtual sensors. In *Advanced Information Networking and Applications (AINA), 2015 IEEE 29th International Conference on*, 332–338 (IEEE, 2015).

[3] Mobile app backend development framework. URL `https://www.progress.com/kinvey`.

[4] Hainmueller, J., Hopkins, D. J. & Yamamoto, T. Causal inference in conjoint analysis: Understanding multidimensional choices via stated preference experiments. *Political analysis* **22**, 1–30 (2014).

[5] Malhotra, N. K. Information load and consumer decision making. *Journal of consumer research* **8**, 419–430 (1982).

[6] Hennig, C. Cluster-wise assessment of cluster stability. *Computational Statistics & Data Analysis* **52**, 258–271 (2007).

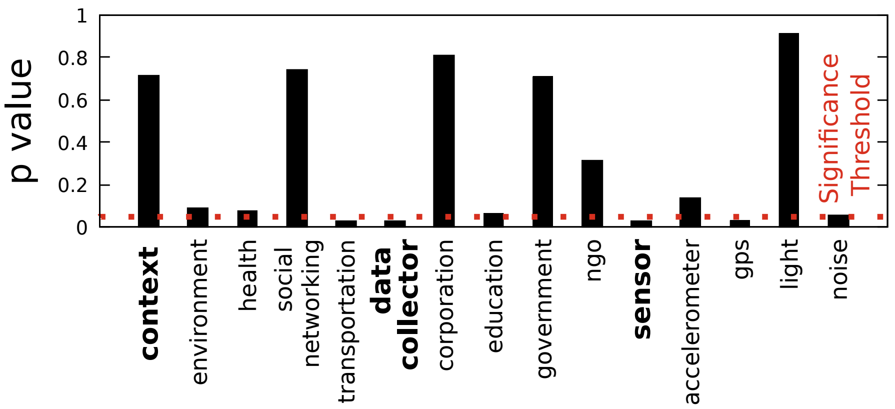

Figure S16: Statistical significance (p values) of the different data-sharing criteria and their elements to explain the five group behaviors in data sharing. Data collectors and sensors are significant ($p < 0.05$ values), in particular the GPS, as well as the transportation context. Moreover, the following data-sharing elements fall close to the significance threshold: environment, and education contexts, accelerometer and noise sensors, and the educational institutes as data collector.

[7] Westin, A. Harris Louis & Associates. Harris-Equifax Consumer Privacy Survey. Tech. Rep., Tech. rep, Conducted for Equifax Inc. 1,255 adults of the US public (1991).

[8] Kumaraguru, P. & Cranor, L. F. *Privacy indexes: a survey of Westin's studies* (Carnegie Mellon University, School of Computer Science, 2005).

Table S16: One-way ANOVA report analysis. Dependent variables: Questions B.9-B12 in Table S4. Independent variable: The privacy change when groups' data-sharing choices are rewarded.

Data Sharing Criterion			Sum of Squares	Degrees of Freedom (*df*)	Mean Squares	F-test	Significance
Sensors		Between	10.745	4	2.686	2.756	0.031
		Within	107.202	110	0.975		
		Total	117.948	114			
	Accelerometer	Between	6.083	4	1.521	1.774	0.139
		Within	94.317	110	0.857		
		Total	100.4	114			
	Location	Between	8.805	4	2.201	2.719	0.033
		Within	89.056	110	0.81		
		Total	97.861	114			
	Light	Between	0.838	4	0.21	0.241	0.914
		Within	95.509	110	0.868		
		Total	96.348	114			
	Noise	Between	14.293	4	3.573	2.384	0.056
		Within	164.873	110	1.499		
		Total	179.165	114			
Data collectors		Between	9.85	4	2.463	2.862	0.027
		Within	94.637	110	0.86		
		Total	104.487	114			
	Corporations	Between	2.019	4	0.505	0.399	0.809
		Within	140.559	111	1.266		
		Total	142.578	115			
	NGOs	Between	5.426	4	1.356	1.199	0.315
		Within	125.566	111	1.131		
		Total	130.991	115			
	Goverments	Between	3.322	4	0.831	0.534	0.711
		Within	172.566	111	1.555		
		Total	175.888	115			
	Educational institutes	Between	7.881	4	1.97	2.27	0.066
		Within	96.36	111	0.868		
		Total	104.241	115			
Context/purpose		Between	2.056	4	0.514	0.532	0.712
		Within	106.24	110	0.966		
		Total	108.296	114			
	Health/fitness	Between	12.013	4	3.003	2.158	0.078
		Within	154.496	111	1.392		
		Total	166.509	115			
	Social networking	Between	1.468	4	0.367	0.495	0.74
		Within	82.325	111	0.742		
		Total	83.793	115			
	Environment	Between	7.606	4	1.901	2.08	0.088
		Within	101.455	111	0.914		
		Total	109.06	115			
	Transport	Between	12.589	4	3.147	2.779	0.03
		Within	125.713	111	1.133		
		Total	138.302	115			

Table S17: Post hoc Tukey's range test ($\alpha = 0.05$) on sensors explaining the privacy change when groups' data-sharing choices are rewarded.

Data Sharing Criterion		Mean Group Differences	Standard Deviation Error	Significance	95% Confidence Interval Lower Bound	Upper Bound
Sensors	reward seekers - privacy ignorants	0.058	0.301	1.0	-0.78	0.89
	privacy neutrals - privacy ignorants	0.779	0.314	0.102	-0.09	1.65
	privacy neutrals - reward seekers	0.721	0.243	0.03	0.05	1.4
	privacy preservers - privacy ignorants	0.511	0.416	0.735	-0.64	1.67
	privacy preservers - reward seekers	0.453	0.366	0.729	-0.56	1.47
	privacy preservers - privacy neutrals	-0.268	0.377	0.953	-1.31	0.78
	reward opportunists - privacy ignorants	0.233	0.325	0.952	-0.67	1.13
	reward opportunists - reward seekers	0.175	0.257	0.96	-0.54	0.89
	reward opportunists - privacy neutrals	-0.546	0.272	0.271	-1.3	0.21
	reward opportunists - privacy preservers	-0.278	0.386	0.952	-1.35	0.79
Accelerometer	reward seekers - privacy ignorants	0.438	0.283	0.536	-0.35	1.22
	privacy neutrals - privacy ignorants	0.703	0.294	0.126	-0.11	1.52
	privacy neutrals - reward seekers	0.266	0.23	0.776	-0.37	0.9
	privacy preservers - privacy ignorants	0.1	0.378	0.999	-0.95	1.15
	privacy preservers - reward seekers	-0.338	0.33	0.844	-1.25	0.58
	privacy preservers - privacy neutrals	-0.603	0.34	0.392	-1.55	0.34
	reward opportunists - privacy ignorants	0.35	0.305	0.78	-0.5	1.2
	reward opportunists - reward seekers	-0.088	0.243	0.996	-0.76	0.59
	reward opportunists - privacy neutrals	-0.353	0.256	0.64	-1.06	0.36
	reward opportunists - privacy preservers	0.25	0.349	0.952	-0.72	1.22
Location	reward seekers - privacy ignorants	0.733	0.275	0.066	-0.03	1.5
	privacy neutrals - privacy ignorants	0.837	0.286	0.033	0.04	1.63
	privacy neutrals - reward seekers	0.103	0.223	0.99	-0.52	0.72
	privacy preservers - privacy ignorants	0.933	0.367	0.089	-0.09	1.95
	privacy preservers - reward seekers	0.2	0.321	0.971	-0.69	1.09
	privacy preservers - privacy neutrals	0.097	0.33	0.998	-0.82	1.01
	reward opportunists - privacy ignorants	0.817	0.296	0.052	0.0	1.64
	reward opportunists - reward seekers	0.083	0.236	0.997	-0.57	0.74
	reward opportunists - privacy neutrals	-0.02	0.248	1.0	-0.71	0.67
	reward opportunists - privacy preservers	-0.117	0.339	0.997	-1.06	0.82
Light	reward seekers - privacy ignorants	0.023	0.285	1.0	-0.77	0.81
	privacy neutrals - privacy ignorants	-0.044	0.296	1.0	-0.87	0.78
	privacy neutrals - reward seekers	-0.067	0.231	0.998	-0.71	0.57
	privacy preservers - privacy ignorants	0.067	0.38	1.0	-0.99	1.12
	privacy preservers - reward seekers	0.043	0.332	1.0	-0.88	0.96
	privacy preservers - privacy neutrals	0.11	0.342	0.998	-0.84	1.06
	reward opportunists - privacy ignorants	-0.192	0.307	0.971	-1.04	0.66
	reward opportunists - reward seekers	-0.215	0.244	0.903	-0.89	0.46
	reward opportunists - privacy neutrals	-0.148	0.257	0.978	-0.86	0.57
	reward opportunists - privacy preservers	-0.258	0.351	0.947	-1.23	0.71
Noise	reward seekers - privacy ignorants	0.272	0.375	0.95	-0.77	1.31
	privacy neutrals - privacy ignorants	0.798	0.389	0.25	-0.28	1.88
	privacy neutrals - reward seekers	0.526	0.304	0.419	-0.32	1.37
	privacy preservers - privacy ignorants	1.267	0.5	0.09	-0.12	2.65
	privacy preservers - reward seekers	0.995	0.436	0.159	-0.22	2.2
	privacy preservers - privacy neutrals	0.469	0.449	0.834	-0.78	1.71
	reward opportunists - privacy ignorants	0.408	0.403	0.849	-0.71	1.53
	reward opportunists - reward seekers	0.136	0.321	0.993	-0.75	1.03
	reward opportunists - privacy neutrals	-0.389	0.338	0.778	-1.33	0.55
	reward opportunists - privacy preservers	-0.858	0.461	0.344	-2.14	0.42

Table S18: Post hoc Tukey's range test ($\alpha = 0.05$) on data collectors explaining the privacy change when groups' data-sharing choices are rewarded.

Data Sharing Criterion		Sum of Squares	Degrees of Freedom (df)	Mean Squares	95% Confidence Interval F-test	Significance
	reward seekers - privacy ignorants	0.502	0.283	0.394	-0.28	1.29
	privacy neutrals - privacy ignorants	0.582	0.295	0.287	-0.24	1.4
	privacy neutrals - reward seekers	0.08	0.229	0.997	-0.55	0.71
	privacy preservers - privacy ignorants	0.911	0.391	0.143	-0.17	2.0
Data collectors	privacy preservers - reward seekers	0.409	0.344	0.757	-0.54	1.36
	privacy preservers - privacy neutrals	0.33	0.354	0.884	-0.65	-1.31
	reward opportunists - privacy ignorants	0.967	0.305	0.017	0.12	1.81
	reward opportunists - reward seekers	0.465	0.242	0.312	-0.21	1.14
	reward opportunists - privacy neutrals	0.385	0.256	0.562	-0.32	1.09
	reward opportunists - privacy preservers	0.056	0.363	1.0	-0.95	1.06
	reward seekers - privacy ignorants	0.286	0.343	0.92	-0.67	1.24
	privacy neutrals - privacy ignorants	0.285	0.358	0.931	-0.71	1,28
	privacy neutrals - reward seekers	-0.001	0.277	1.0	-0.77	0.77
	privacy preservers - privacy ignorants	0.433	0.459	0.879	-0.84	1.71
Corporations	privacy preservers - reward seekers	0.147	0.4	0.996	-0.96	1.26
	privacy preservers - privacy neutrals	0.148	0.413	0.996	-1.0	1.29
	reward opportunists - privacy ignorants	0.442	0.37	0.756	-0.59	1.47
	reward opportunists - reward seekers	0.156	0.293	0.984	-0.66	0.97
	reward opportunists - privacy neutrals	0.157	0.311	0.987	-0.7	1.02
	reward opportunists - privacy preservers	0.008	0.424	1.0	-1.17	1.18
	reward seekers - privacy ignorants	0.374	0.324	0.778	-0.53	1.27
NGOs	privacy neutrals - privacy ignorants	0.607	0.338	0.382	-0.33	1.54
	privacy neutrals - reward seekers	0.233	0.262	0.9	-0.49	0.96
	privacy preservers - privacy ignorants	0.8	0.434	0.355	-0.4	2.0
	privacy preservers - reward seekers	0.426	0.378	0.792	-0.62	1.47
	privacy preservers - privacy neutrals	0.193	0.39	0.988	-0.89	1.27
	reward opportunists - privacy ignorants	0.317	0.35	0.895	-0.65	1.29
	reward opportunists - reward seekers	-0.057	0.277	1.0	-0.83	0.71
	reward opportunists - privacy neutrals	-0.29	0.293	0.86	-1.1	0.52
	reward opportunists - privacy preservers	-0.483	0.4	0.747	-1.59	0.63
	reward seekers - privacy ignorants	0.263	0.38	0.958	-0.79	1.32
Goverments	privacy neutrals - privacy ignorants	0.552	0.397	0.635	-0.55	1.65
	privacy neutrals - reward seekers	0.289	0.307	0.881	-0.56	1.14
	privacy preservers - privacy ignorants	0.4	0.509	0.934	-1.01	1.81
	privacy preservers - reward seekers	0.137	0.443	0.998	-1.09	1.37
	privacy preservers - privacy neutrals	-0.152	0.457	0.997	-1.42	1.12
	reward opportunists - privacy ignorants	0.375	0.41	0.891	-0.76	1.51
	reward opportunists - reward seekers	0.112	0.325	0.997	-0.79	1.01
	reward opportunists - privacy neutrals	-0.177	0.344	0.986	-1.13	0.78
	reward opportunists - privacy preservers	-0.025	0.496	1.0	-1.33	1.28
	reward seekers - privacy ignorants	0.528	0.284	0.346	-0.26	1.32
Educational institutes	privacy neutrals - privacy ignorants	0.237	0.296	0.93	-0.58	1.06
	privacy neutrals - reward seekers	-0.291	0.23	0.711	-0.93	0.35
	privacy preservers - privacy ignorants	1.033	0.38	0.058	-0.02	2.09
	privacy preservers - reward seekers	0.505	0.331	0.548	-0.41	1.42
	privacy preservers - privacy neutrals	0.797	0.342	0.143	-0.15	1.74
	reward opportunists - privacy ignorants	0.342	0.307	0.799	-0.51	1.19
	reward opportunists - reward seekers	-0.186	0.243	0.939	-0.86	0.49
	reward opportunists - privacy neutrals	0.105	0.257	0.994	-0.61	0.82
	reward opportunists - privacy preservers	-0.692	0.351	0.286	-1.66	0.28

Table S19: Post hoc Tukey's range test ($\alpha = 0.05$) on data-sharing context/purpose explaining the privacy change when groups' data-sharing choices are rewarded.

Data Sharing Criterion		Sum of Squares	Degrees of Freedom (df)	Mean Squares	95% Confidence Interval F-test	Significance
Context/purpose	reward seekers - privacy ignorants	0.119	0.3	0.995	-0.71	0.95
	privacy neutrals - privacy ignorants	0.17	0.313	0.982	-0.7	1.04
	privacy neutrals - reward seekers	0.051	0.242	1.0	-0.62	0.72
	privacy preservers - privacy ignorants	0.511	0.414	0.732	-0.64	1.66
	privacy preservers - reward seekers	0.392	0.364	0.819	-0.62	1.4
	privacy preservers - privacy neutrals	0.341	0.375	0.893	-0.7	1.38
	reward opportunists - privacy ignorants	0.317	0.323	0.864	-0.58	1.21
	reward opportunists - reward seekers	0.197	0.256	0.939	-0.51	0.91
	reward opportunists - privacy neutrals	0.147	0.271	0.983	-0.61	0.9
	reward opportunists - privacy preservers	-0.194	0.384	0.987	-1.26	0.87
Health/fitness	reward seekers - privacy ignorants	0.765	0.36	0.216	-0.23	1.76
	privacy neutrals - privacy ignorants	1.064	0.375	0.042	0.02	2.1
	privacy neutrals - reward seekers	0.299	0.291	0.841	-0.51	1.11
	privacy preservers - privacy ignorants	0.833	0.482	0.42	-0.5	2.17
	privacy preservers - reward seekers	0.068	0.419	1.0	-1.09	1.23
	privacy preservers - privacy neutrals	-0.231	0.433	0.984	-1.43	0.97
	reward opportunists - privacy ignorants	0.925	0.388	0.128	-0.15	2.0
	reward opportunists - reward seekers	0.16	0.308	0.985	-0.69	1.01
	reward opportunists - privacy neutrals	-0.139	0.326	0.993	-1.04	0.76
	reward opportunists - privacy preservers	0.092	0.444	1.0	-1.14	1.32
Social networking	reward seekers - privacy ignorants	0.318	0.263	0.746	-0.41	1.05
	privacy neutrals - privacy ignorants	0.271	0.274	0.859	-0.49	1.03
	privacy neutrals - reward seekers	-0.46	0.212	0.999	-0.64	0.54
	privacy preservers - privacy ignorants	0.433	0.352	0.732	-0.54	1.41
	privacy preservers - reward seekers	0.116	0.306	0.996	-0.73	0.96
	privacy preservers - privacy neutrals	0.162	0.316	0.986	-0.71	1.04
	reward opportunists - privacy ignorants	0.3	0.283	0.827	-0.49	1.09
	reward opportunists - reward seekers	-0.018	0.225	1.0	-0.64	0.61
	reward opportunists - privacy neutrals	0.029	0.238	1.0	-0.63	0.69
	reward opportunists - privacy preservers	-0.133	0.324	0.994	-1.03	0.77
Environment	reward seekers - privacy ignorants	0.486	0.292	0.459	-0.32	1.29
	privacy neutrals - privacy ignorants	0.657	0.304	0.202	-0.19	1.5
	privacy neutrals - reward seekers	0.172	0.236	0.95	-0.48	0.83
	privacy preservers - privacy ignorants	0.933	0.39	0.125	-0.15	2.02
	privacy preservers - reward seekers	0.447	0.34	0.681	-0.49	1.39
	privacy preservers - privacy neutrals	0.276	0.351	0.934	-0.7	1.25
	reward opportunists - privacy ignorants	0.767	0.315	0.113	-0.11	1.64
	reward opportunists - reward seekers	0.281	0.249	0.792	-0.41	0.97
	reward opportunists - privacy neutrals	0.109	0.264	0.994	-0.62	0.84
	reward opportunists - privacy preservers	-0.167	0.36	0.99	-1.16	0.83
Transport	reward seekers - privacy ignorants	0.351	0.325	0.816	-0.55	1.25
	privacy neutrals - privacy ignorants	0.908	0.338	0.063	-0.03	1.85
	privacy neutrals - reward seekers	0.557	0.262	0.218	-0.17	1.28
	privacy preservers - privacy ignorants	0.767	0.434	0.399	-0.44	1.97
	privacy preservers - reward seekers	0.416	0.378	0.807	-0.63	1.46
	privacy preservers - privacy neutrals	-0.141	0.39	0.996	-1.22	0.94
	reward opportunists - privacy ignorants	0.875	0.35	0.098	-0.1	1.85
	reward opportunists - reward seekers	0.524	0.277	0.329	-0.25	1.29
	reward opportunists - privacy neutrals	-0.033	0.294	1.0	-0.85	0.78
	reward opportunists - privacy preservers	0.108	0.401	0.999	-1.0	1.22
